# *DefectTrack*: a deep learning-based multi-object tracking algorithm for quantitative defect analysis of in-situ TEM videos in real-time

**DOI:** 10.1038/s41598-022-19697-1

**Published:** 2022-09-20

**Authors:** Rajat Sainju, Wei-Ying Chen, Samuel Schaefer, Qian Yang, Caiwen Ding, Meimei Li, Yuanyuan Zhu

**Affiliations:** 1 Department of Materials Science and Engineering, University of Connecticut, Storrs, CT 06269 USA; 2 Nuclear Science and Engineering Division, Argonne National Laboratory, Lemont, IL 60439 USA; 3 Department of Computer Science and Engineering, University of Connecticut, Storrs, CT 06269 USA

**Keywords:** Energy science and technology, Engineering, Materials science

## Abstract

In-situ irradiation transmission electron microscopy (TEM) offers unique insights into the millisecond-timescale post-cascade process, such as the lifetime and thermal stability of defect clusters, vital to the mechanistic understanding of irradiation damage in nuclear materials. Converting in-situ irradiation TEM video data into meaningful information on defect cluster dynamic properties (e.g., lifetime) has become the major technical bottleneck. Here, we present a solution called the *DefectTrack*, the first dedicated deep learning-based one-shot multi-object tracking (MOT) model capable of tracking cascade-induced defect clusters in in-situ TEM videos in real-time. *DefectTrack* has achieved a Multi-Object Tracking Accuracy (MOTA) of 66.43% and a Mostly Tracked (MT) of 67.81% on the test set, which are comparable to state-of-the-art MOT algorithms. We discuss the MOT framework, model selection, training, and evaluation strategies for in-situ TEM applications. Further, we compare the *DefectTrack* with four human experts in quantifying defect cluster lifetime distributions using statistical tests and discuss the relationship between the material science domain metrics and MOT metrics. Our statistical evaluations on the defect lifetime distribution suggest that the *DefectTrack* outperforms human experts in accuracy and speed.

## Introduction

In-situ transmission electron microscopy (TEM) is a powerful characterization tool that allows direct observations of dynamic changes in materials under technically relevant working conditions in real-time^1^. Specifically, the Intermediate Voltage Electron Microscopy (IVEM)—Tandem Facility at Argonne National Laboratory (ANL) offers unique in-situ TEM studies under simulating nuclear reactor environments with synergistic effects of irradiation, temperature, and stress^2^. It is well known that irradiation (by high-energy particles such as neutrons or ions) can displace atoms, leading to the continual production of cascades of defects. The subsequent agglomeration of such defects produces nanometer-size clusters^3^. Therefore, a clear understanding of the dynamic evolution of cascade-induced defect clusters plays a vital role in developing a comprehensive understanding of the mechanisms of irradiation damage for core and structural nuclear reactor materials. In particular, the lifetime of unstable defect clusters is one of the decisive factors that governs the equilibrium defect density and the onset of void swelling^4^. However, the laborious manual defect analysis is a major technical bottleneck in in-situ irradiation characterization. This technical bottleneck has become an increasingly pressing limitation for in-situ irradiation TEM video interpretation, especially as fast cameras are beginning to produce an ‘avalanche’ of TEM video data. For instance, ANL’s IVEM can collect 10-megapixel images at up to 313 frames per second (FPS), producing gigabytes-to-terabytes of in-situ TEM videos in a single irradiation experiment. Therefore, the lack of consistent and reproducible defect analysis methods has profoundly hindered our understanding of the dynamic processes of defect clusters evolution and slowed irradiation mechanism discovery.

Filling key gaps in the knowledge base of cascade-induced defect clusters and solving the above challenging technical bottleneck requires novel approaches. In recent years, deep learning has demonstrated breakthroughs in computer vision-related tasks for automated image and video processing^5–7^, and in scientific research (e.g., cell tracking^8,9^, controlling plasma in tokamak^10^). To date, most deep learning-based computer vision applications to the materials science domain are mainly focused on relatively simple tasks like classification^11,12^, object detection^13–15^, and semantic segmentation^16–19^ of microscopy image data. For high-throughput tracking of cascade-induced defect clusters and their dynamic evolution in in-situ irradiation TEM videos, deep learning-based multi-object tracking (MOT) algorithm^20^ is a promising approach, yet, has not been realized. MOT is defined as the task of predicting the trajectories of the objects of interest in videos or image sequences. The current tracking application is restricted to dislocation loop tracking^14^ and nanoparticle tracking^21,22^ that use two-compute intensive separate models for object detection and tracking. The tracking model usually contains a traditional computer vision algorithm with a slow rigid feature Re-ID and association method. This two-model strategy is not conducive to developing real-time tracking systems as the total runtime is the sum of the runtime of the two separate models, where Re-ID model is separately applied to every bounding box detection. To enable high-speed real-time tracking, it is necessary to utilize deep learning-based one-shot (simultaneous detection and tracking using a shared network) MOT models^23,24^ that have demonstrated effective fast-tracking of everyday objects.

**Figure 1:**
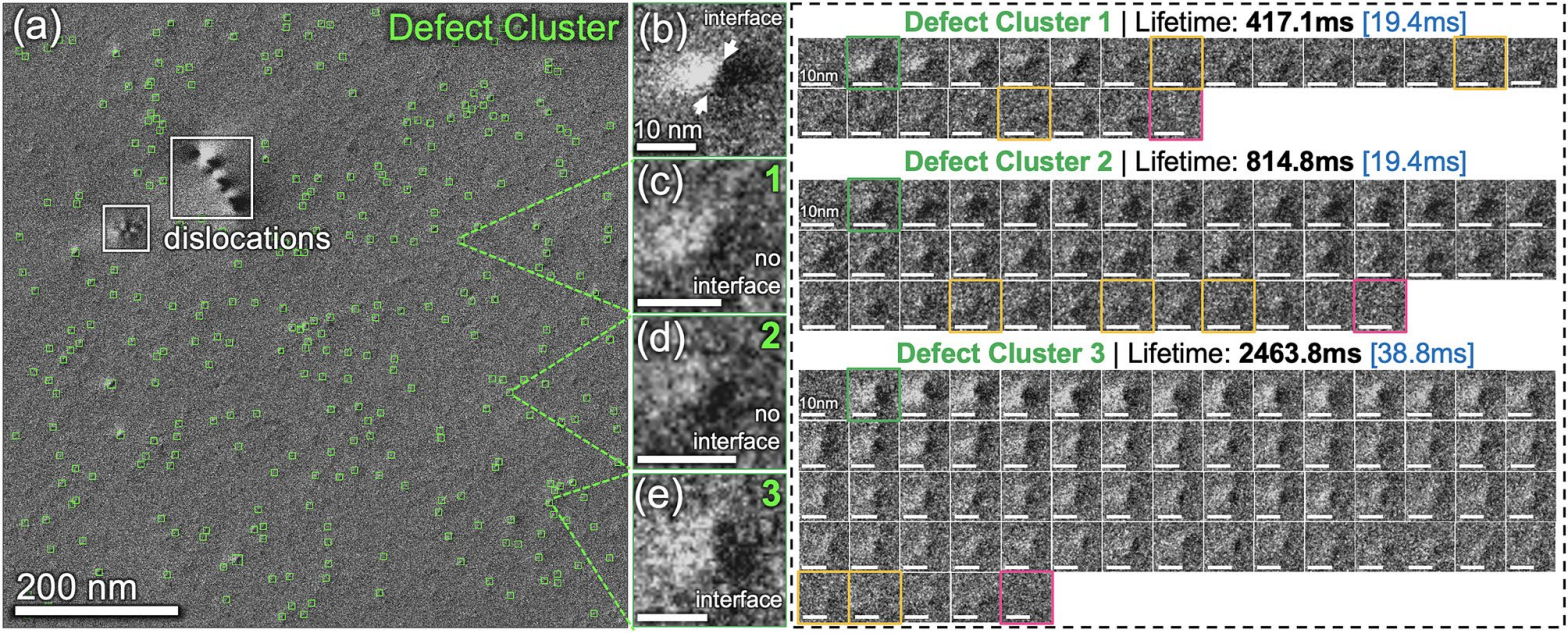
Representative defect clusters in a snapshot of an in-situ TEM video acquired during 1 MeV Kr^2+^ ions irradiation of a pure nickel at 700 °C. (**a**) An in-situ TEM image frame with 320 defect clusters (green boxes) and dislocations (white boxes). (**b–e**) Individual defect clusters exhibit different contrasts and representations. (right). In-situ TEM image series of three selected defect clusters with different lifetimes. The time interval between frames marks in blue. In these sequences, the green outlined frames indicate when a defect cluster became visible; the red frames outline its annihilated, and the yellow frames mark when the defect clusters suddenly appear to be indistinguishable from the background.

Fast in-situ TEM videos of cascade-induced defect clusters pose particular challenges to this object tracking task, due to the presence of small defects^25^ with inhomogeneous and evolving feature representations. Fig. 1 showcases cascade-induced defect clusters in an in-situ TEM video snapshot acquired during 1 MeV Kr^2+^ ions irradiation of a pure nickel at 700° C. This TEM frame contains a high density of defect clusters (320 counts) that are relatively small (an averaged size of 7.66 nm or 20 pixels), exhibiting a wide range of feature representations, contrast, and lifetimes. For example, the defect clusters in Fig. 1b,e show a sharp interface with a black-and-white lobe pattern, while in Fig. 1c,d, the interface is not clear. The black-white lobe contrast originates from the asymmetrical strain field of defect clusters produced by irradiation. The time-series of the individual Defect Cluster#1–3 show that most defect clusters initially exhibit a sharp interface and, in time, their contrast faded monotonically and thus their interfaces become unclear. Usually, the defect contrast is much fainter and the size is smaller at the end of their lifetime. Moreover, there are cases where the defect cluster suddenly changes its appearance and is indistinguishable from the background (outlined by yellow). To quantify the lifetime of defect clusters, it is necessary to keep track of the formation, evolution, and annihilation of each defect clusters throughout an irradiation experiment. Altogether, the small size, a wide range of feature representations, varying lifetimes, and nonlinear changes in the defect contrast make online tracking of defect clusters a challenging task.

In this study, we developed the *DefectTrack*, the first one-shot end-to-end deep learning-based MOT model capable of real-time detecting and tracking of nano-sized defect clusters in in-situ irradiation TEM videos. We established an MOT in-situ TEM video training dataset using the standard tracking annotation protocol. Several strategies were tested to identify the optimal model and training strategy to track the defects clusters in in-situ TEM video data with high fidelity. We performed both the standard MOT metrics evaluation and the materials science-relevant defect lifetime distribution evaluation, to thoroughly assess the model performance. For materials science domain evaluation, the performance of our *DefectTrack* in predicting the defect cluster lifetime distribution was compared with a group of human experts using statistical tests. Further, we inspected the relationship between the MOT metrics and the lifetime distribution statistical test metrics. These evaluations suggest that MOT holds great potential for robust, reliable, and fast defect tracking in in-situ TEM videos.

## Methods

### In-situ irradiation TEM imaging

An annealed nickel TEM specimen was irradiated at 700ºC with 1 MeV krypton ions with a flux of 1.3 × 10^12^ ions/(cm^2^·s) in the IVEM-Tandem facility at ANL, where the microstructural evolution under irradiation was observed in-situ with a Hitachi-9000 TEM operated at 300 kV. During irradiation, the microstructure was video recorded in the dark-field mode with 103 FPS and an image size of 2048 pixels by 2048 pixels. The pixel resolution is 0.38 nm per pixel. The imaging condition was a two-beam dynamical condition with g = 200 near the [011] zone axis using a Gatan OneView camera. A detailed description of the experiment can be found in the authors’ previous paper^26^.

### Benchmark dataset

**Figure 2:**
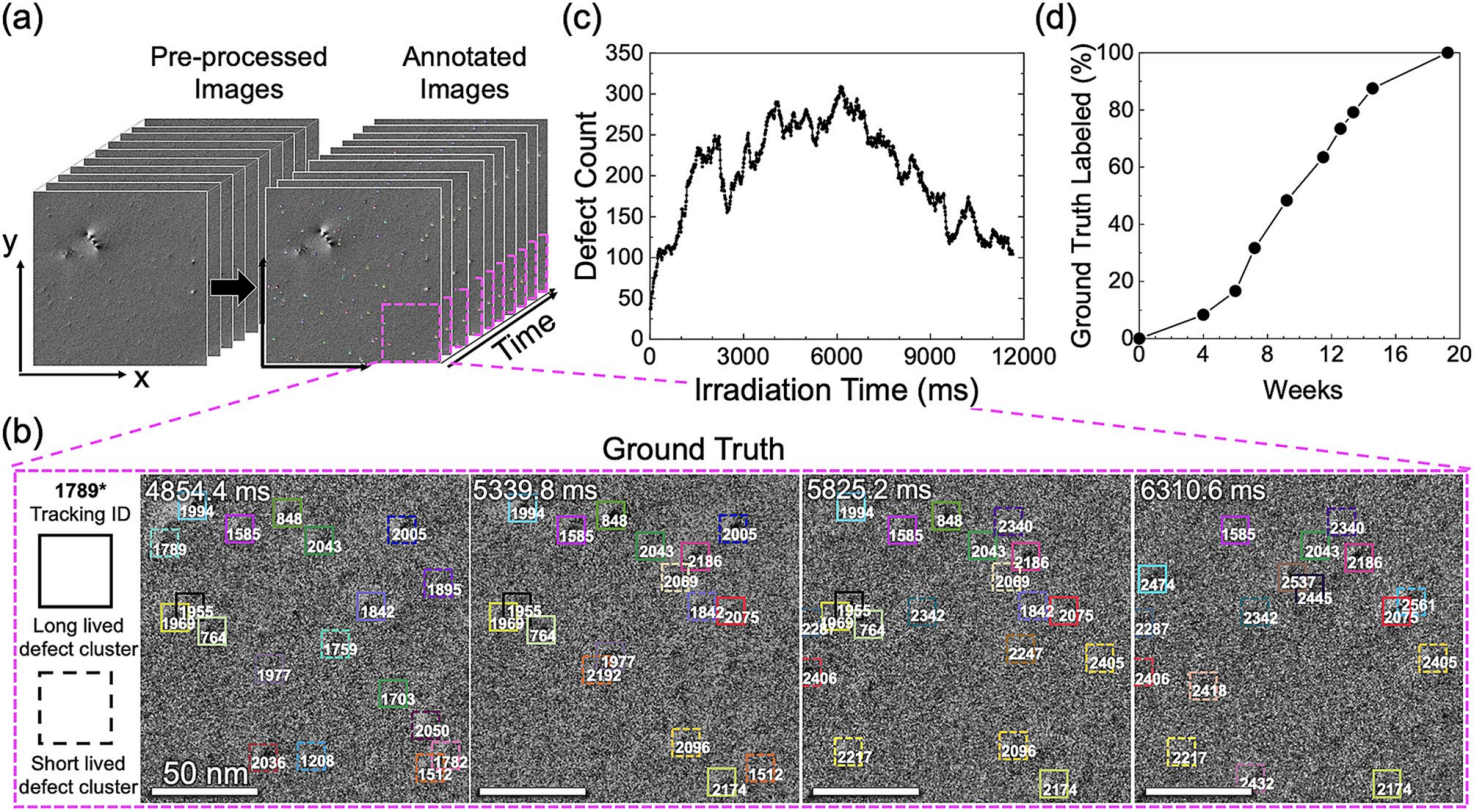
An overview of the ground truth annotation, defect count quantification, and labeling cost. (**a**) An in-situ irradiation TEM video of 1200 frames (9.7 ms/frame) was manually annotated. (**b**) Illustration of the annotation performed on example video frames. Both long-lived (solid lines) and short-lived (dashed lines) defects are shown with their unique tracking ID. Defect clusters with a lifetime longer than 980.5 ms are considered long-lived. (**c**) Defect count as a function of irradiation time quantified directly from the annotation. (**d**) Time spent on ground truth manual annotation and validation.

Ground truth annotation was carried out in VGG Image Annotator (VIA)^27^. Prior to the annotation, image pre-processing, including drift correction and image normalization, was performed on the entire video (Supplementary Sect. 1). Unlike conventional object detection annotation, the MOT annotation requires assigning and maintaining unique tracking IDs.

for each defect cluster (bounding box) throughout its lifetime. Figs. 2a,b showcase examples of our ground truth annotation of a 1200-frame (2048 × 2048 pixels) TEM video, with each defect cluster labeled and tracked by a uniquely colored bounding box and tracking ID. Fig. 2c shows the total number of defects as a function of irradiation time in the dataset. Three researchers experienced in radiation defect analysis worked meticulously and iteratively in assigning and validating each label. The ground truth development took about 20 weeks (Fig. 2d) and successfully detected 243,052 defect clusters, with 4279 unique defect clusters (i.e., defects with the same unique ID) tracked throughout the entire video. This benchmark dataset includes annotated in-situ irradiation TEM video following the standard protocol outlined for MOTChallenge^28^. One important thing to note is that the motion of defect clusters is almost negligible (i.e., only about 2.5 ± 1.1 pixels). Lastly, to facilitate the training of *DefectTrack*, this annotated dataset was divided in the spatial dimension into eight video sequences of 1200 frames 1024 × 512 pixels (Supplementary Sect. 2). While dividing an entire video into pieces leads to incomplete defect clusters usually located at the frame edges, such defects constituted < 1% of the total defects and thus were omitted from the labeling. The resulting eight sequences have a similar number of defect clusters and defect cluster tracks (Fig. S2b).

### Model network

**Figure 3:**
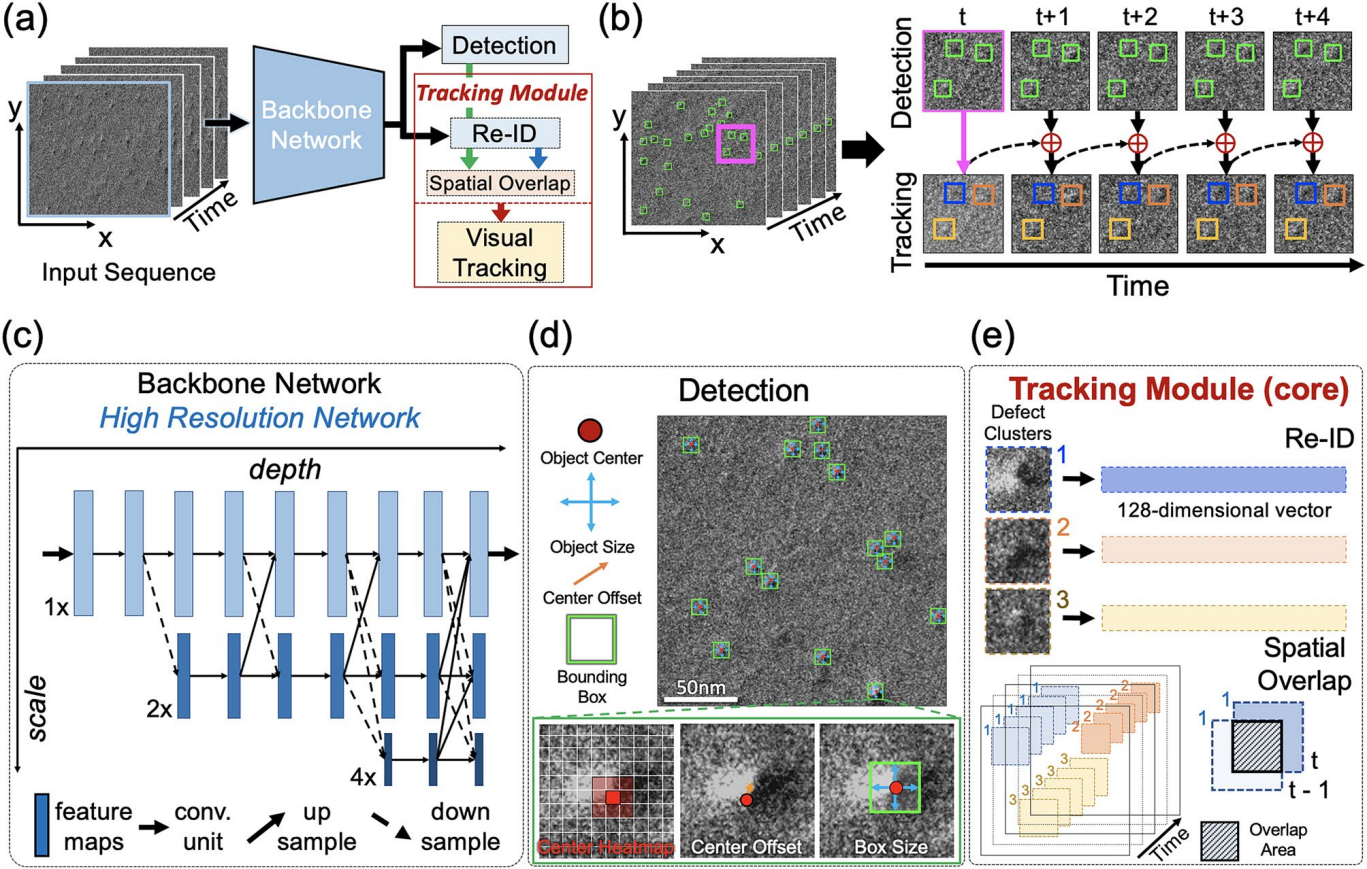
Overview of our *DefectTrack* model network. (**a**) *DefectTrack* architecture. (**b**) Schematics of how *DefectTrack* achieves tracking via detection and tracklets association. (**c**) The neural network architecture of the backbone network. (**d**) *DefectTrack*’s detection branch. (**e**) *DefectTrack*’s core tracking module.

Fig. 3a presents an overview of our *DefectTrack* model architecture. We customized the FairMOT^24^ framework to track defect clusters and obtain a statistically significant measurement of defect cluster lifetime distribution in in-situ irradiation TEM videos. Our *DefectTrack* utilizes a simple network structure consisting of a backbone network with two branches: (1) the detection branch for detecting defect clusters and (2) the Re-ID branch to extract the re-ID features for each detected defect cluster. The predictions from these two branches are passed onto the tracking module for the online track association. As illustrated in Fig. 3b, the *DefectTrack* uses information from past frames to make predictions on the current frame and thus achieve tracking. Based on the detections in the first frame (highlighted in magenta), several tracks are initialized. Then the detected defect clusters in the subsequent frames are associated with the existing tracklets. It is worth mentioning that our *DefectTrack* is not a simple detection model; instead, it is a tracking model.

#### Detection and Re-ID

In this section, we first describe the backbone network, the detection branch, and the re-ID branch. We employed a customized High-Resolution Network^29^ (HRNet-W18) as the backbone network (Fig. 3c). Compared to encoder-decoder methods, which adopt a high-resolution recovery process, the advantage of HRNet-W18 is that it maintains a high-resolution representation throughout the process^29^. HRNet-w18 also outperformed our initial backbone-network candidates (Fig. S3a). The high-resolution feature map outputs make it easier to identify the ‘small’ defect clusters that exist in the benchmark dataset. We changed the model input image size from *W* × *H* × 3 (*W* = width, *H* = height in pixels; 3 for RGB channels) pixels to *W’* × *H’* × 1 (*W’* = 8/15* W, H’* = *H*/2; 1 channel for grayscale images). Since the in-situ TEM video frames are grayscale images, the convolution kernel size of the first layer was changed from 3 × 3 × 3 to 3 × 3 × 1 as the original algorithm is built for prediction on everyday RGB images with MOTChallenge standardized image sizes. *DefectTrack* performs detection and re-ID^30,31^ on high-resolution feature maps of stride four meaning the output feature map size is *W’/4* × *H’/4.* Traditional object detectors use a large output stride of 16^32^. Different from conventional detection methods, the detection is performed by an anchor-free method that formulates object detection as a key-point estimation problem^33^. The high-resolution feature map output from the backbone network is appended with three parallel heads in the detection branch (Fig. 3d) to estimate the object-center, center-offsets, and box-sizes^24,33^. These three pieces of information are combined to predict the defect cluster position and size. Lastly, non-maximum suppression^33^ and confidence thresholding were applied to make the final prediction. The object confidence score is calculated based on the object heatmap score^33^. For details of the detection framework see^33^. For re-identification (Re-ID), the goal is to generate re-ID features that have similar affinity for the same defect cluster across time and lower affinity for different ones^30^. To generate the re-ID features, a convolution layer with 128 kernels was applied to the high-resolution output from the backbone network to extract a 128-dimensional vector for each position in the heatmap (*W’/4* × *H’/4*). *DefectTrack* learns re-ID features like a classification task and essentially encapsulates the appearance of defect clusters that is updated in successive frames^24^. As shown in Fig. 3e, each detected defect cluster is described by a re-ID vector, and by comparing the similarity of re-ID vectors of the detected defect cluster in the current frame with the one in the previous frames using cosine distance^24^, defect clusters with the same identity can be associated across frames.

#### Multiple object tracking

*DefectTrack*’s tracking function is realized by utilizing defect detection, re-ID features, bounding box spatial overlap, and visual tracking. To perform tracking, we first initialize the tracklets based on the detections in the first frame. Then, the operation of the tracking module can generally be divided into two levels. Fig. 3e illustrates the first level of tracking (also the core tracking method), which links the defect clusters into tracks based on the cosine distances between the Re-Id features and the spatial overlap via bipartite matching^34^. Here, spatial overlap (Fig. 3e) is the intersection over the union (IoU) of two bounding boxes at frames *t-1* and *t*. In addition, Kalman filter^35^ is used to predict the location of defect clusters in future frames and obtain appropriate defect tracklets. Overall, we combine re-ID features, spatial overlap, and Kalman filter to perform the first-level multi-object tracking. For further details of the online association step see^24^.

**Figure 4:**
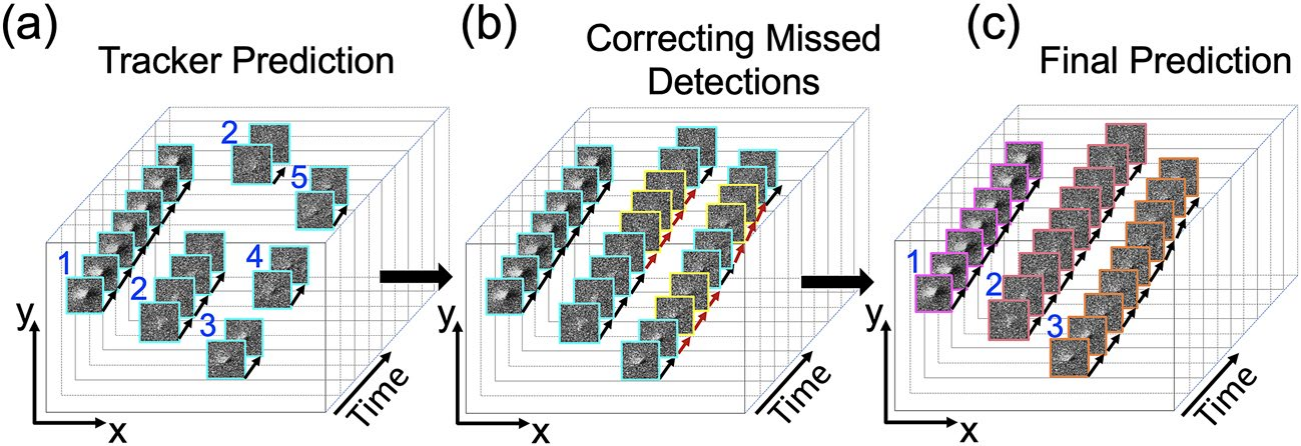
Schematics of the second level of tracking with a visual tracker. (**a**) An example of tracker prediction after the first level of tracking. Some defect cluster tracks are fragmented mainly due to missed detections (False Negatives) and it increases the chances of identity switches (ID SW: ID#3, 4, 5 are assigned to the same defect cluster). (**b**) The correction of missed detections by the visual tracker. Corrected missed detections are outlined in yellow. (**c**) The final output of *DefectTrack* shows uninterrupted and accurate tracking of three defect clusters with unique IDs throughout their lifetime.

The second level of tracking, which we refer to here as visual tracking, was added to mitigate missed detections, which are known to be challenging to handle and often lead to poor tracking performances^36^. This problem of missed detection can be severe when the object of interest is small (< 32 × 32 pixels)^25^. Our defect clusters have an average size of 20 × 20 pixels. Additionally, defects that undergo sudden appearance changes are likely to be missed by the detector, no re-ID feature is generated, and fails to be tracked further. The original implementation trained to track humans failed to capture the characteristics of the cascade-induced defect clusters, such as decreases in contrast and size over their lifetime, sudden termination, shot noise, and dynamic changes in local TEM diffraction conditions. Thus, we designed a second level of tracking inspired by Bochinski et al.’s visual tracking^36^. Here, visual tracking is performed only in the forward direction. Fig. 4 demonstrates the basic principle of the visual tracking process. In Fig. 4a, tracking predictions of defect cluster ID#2 is fragmented (Fig. S5) due to missed detections, and for ID#3, the same defect cluster is identified with multiple IDs# 3, 4, and 5 also called ID Switch (IDSw) (Fig. S5b). To correct such cases, the visual tracker intelligently fills in the missed detections resolving IDSw by propagating the missing information into future frames. During tracking visual tracker is activated if a defect cluster in the current frame does not satisfy a spatial overlap threshold (σ_IOU_) of 0.8 with previous detections. If this criterion is met within *t*_VIOU_ (10) frames, the tracks are merged, and missed detections are interpolated. Otherwise, visual tracking ends for that defect cluster. As shown in Fig. 4b, this tracking procedure retrieves the missed detections, enabling the tracking of long-lived defect clusters and reducing the number of IDSw and track fragmentations. Collectively, the two levels of tracking resulted in uninterrupted and accurate defect cluster tracks (Fig. 4c). Moreover, we applied two additional criteria to the visual tracker to mitigate the problem of false-positive tracks^36,37^. First, a minimum track length criterion was applied to remove defect cluster tracks that last fewer than *t*_min_ (2) frames. These short tracks are primarily due to false-positive detections and could skew the defect clusters’ lifetime distribution. Second, each defect cluster track is required to contain at least one detection with high detection confidence score of σ_h_ (0.6).

*DefectTrack* was trained on the in-situ TEM video data, and the best model was selected based on the performance of the validation sets using the *k*-fold (eight-fold in our case) cross-validation technique^38^. We report the test set performance and further evaluate the model stability^39^ among different combinations of the divided dataset. Details of model selection, training procedure, and implementation see Supplementary Sect. 3.

### Performance evaluation and lifetime distribution assessment

In this work, we evaluated detection using the four commonly used detection metrics: precision, recall, Average Precision (AP), and F1-score. The tracking performance was assessed using the widely accepted MOT metrics^40–42^. In particular, we used the evaluation metrics such as Mostly Tracked (MT), Multi-Object Tracking Accuracy (MOTA), Multi-Object Tracking Precision (MOTP), IDF1, and ID switches (IDSw) to assess the tracking performance metrics. Among these, MT, MOTA, and IDF1 were monitored to inform tracking performance during training. For details of the detection and MOT metrics, see Supplementary Sect. 4. In addition to the machine learning MOT metrics above, we compared the predicted lifetime distribution (by the *DefectTrack* and by Human Experts) with the ground truth lifetime distribution and evaluated the similarity of the two sets companions using statistical tests, including the Kolmogorov-Smirnov^43^ and the Chi-Square^44^ tests. The four experienced human experts who participated in this study have more than 5 years of experience in radiation defect analysis. The description and interpretation of the two statistical tests are detailed in Supplementary Sect. 5.

## Results and discussion

### Detection of defect clusters

Robust and accurate detection is essential to reliable tracking of defect clusters in a video. Instead of training the entire *DefectTrack* directly on its tracking function, we found that training the network first on defect cluster detection promoted the overall performance and subsequently reduced the tracking training complexity. Specifically, after obtaining a preliminary detection model (Supplementary Sect. 3), we worked on identifying the sources of detection errors and mitigated their effects. Within a couple of iterations, we achieved an excellent detection performance. Below, we first report the performance of the *DefectTrack’s* standalone detection (no detection information from past frames is used to make detections on the future frames), and then discuss the sources of detection errors and their mitigation.

Considering that the calculation of detector performance depends on the IoU threshold, rather than taking the IoU = 0.5 as used in the computer vision challenges^45^, we surveyed the AP and F1 as a function of IoU (Fig. S4). For our small defect clusters (lower total area), the computed IoU is sensitive to the bounding box parameter prediction (i.e., position and size). A minute but reasonable shift in the box parameters by a couple of pixels significantly alter the IoU value, affecting AP and F1. F1 and AP decrease with increasing IoU threshold (Fig. S4). In this work, we set the IoU threshold to 0.3 for two reasons: (1) a lower IoU threshold works better for the small defect clusters in our in-situ TEM videos, and (2) it accommodates better the evolving and sometimes not well-specified defect size as the defect clusters are evolving during ion irradiation (Fig. 1). Table 1 summarizes the standalone detection performance of the *DefectTrack* over the eight test sets: AP of 78.41 ± 4.16%, F1-score of 79.38 ± 3.33%, Precision of 77.97 ± 4.17%, and Recall of 80.99 ± 4.023%. While most current SOTA models on the MOTChallenge^28^ do not report their standalone detection performance, we interpolated their detection accuracy from their tracking results, the F1-scores of the top two MOT models GMOTv2 and TransCenter^46^ are 88.32% and 78.90%, respectively^7^. Despite the interpolated detection performance of tracker being much higher than the actual standalone detection performance, as they have access to information from the past video frames to make better detections, the F1-score of our standalone detector (F1 = 79.38%) is comparable to the interpolated detection performance of these SOTA models. Moreover, when compared with dedicated detectors, which are applied to the “small objects”^25^, our *DefectTrack’s* standalone detector (AP = 78.41%) well outperforms the recent FairMOT detectors using HRNet-w18 (AP = 51.10%), and the DLA-34 (AP = 46.80%)^24^.

**Table 1:** *DefectTrack*’s standalone detection performance.

Model detection performance	F1 ↑	AP ↑	Precision ↑	Recall ↑
Eight-fold cross-validation	79.38 ± 3.33	78.41 ± 4.16	77.97 ± 4.17	80.99 ± 4.02

Average detection performance sets for eight-fold cross-validation and the overall standard deviation across all image sequences are presented here. Note that up arrows indicate that the higher the score the better the model performance. For details of the detection metrics, see Supplementary Sect. 4.

**Figure 5:**
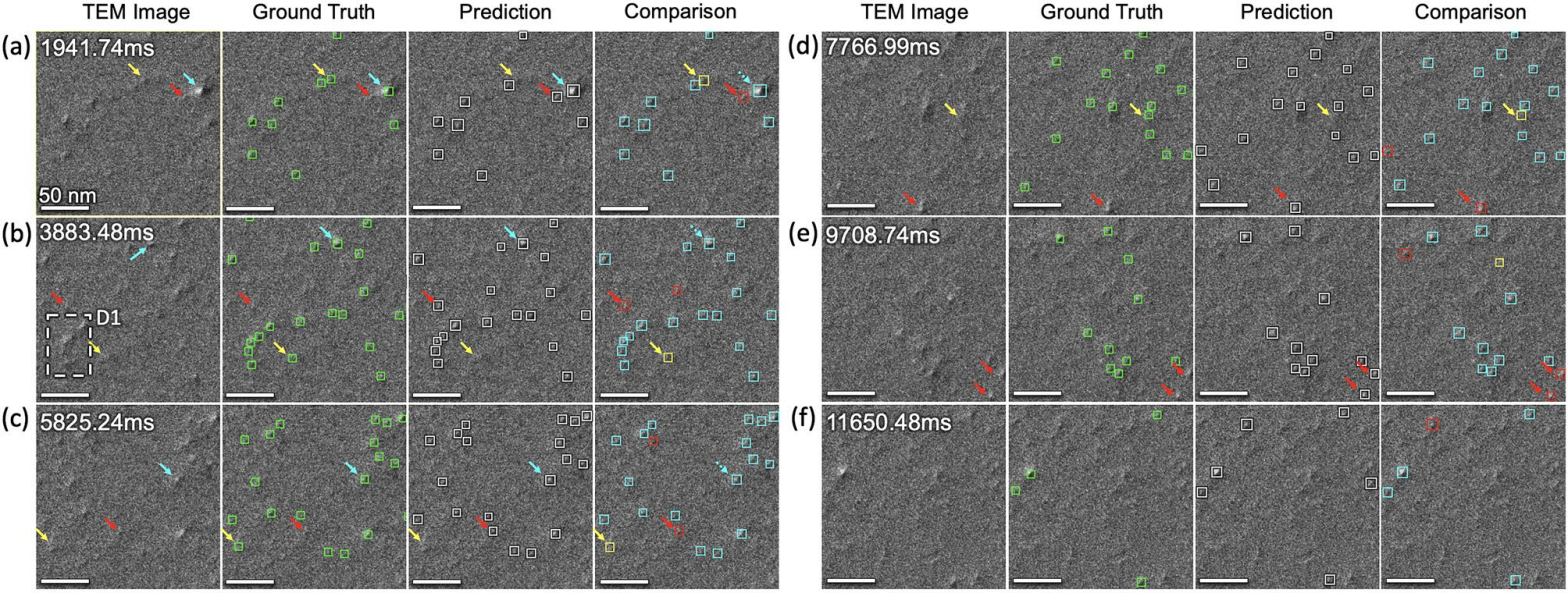
*DefectTrack’s* standalone detector performance. (**a–f**) Examples of the detector performance visualized in selected in-situ TEM video frames with color-coded confusion box-map comparison: true positive (turquoise), false positive (red), and false negative (yellow). Detection is presented here at video frames with a time interval of 1941.7 ms. Turquoise arrows indicate defect clusters with high detection confidence. Yellow arrows mark representative defect clusters with considerably weak contrast and are occasionally missed by the detector. Red arrows mark the misidentified defect clusters.

To inspect *DefectTrack*’s detection performance, we generated a color-coded confusion matrix (TP in turquoise, FP in red, and FN in yellow) for each predicted defect cluster in TEM video frames. Figs. 5a–e illustrate examples of our detections visualized directly on the video frames. Remarkably, the detector correctly identifies most small defect clusters (TP in Turquoise) with different appearances (e.g., defects with a visible interface and no clear interface in Fig. 1). We think this detection performance is attributed to the combination of training optimizations carried out to achieve the lowest generalization error and the use of the HRNet that utilizes higher resolution feature maps that are spatially precise and semantically robust in detecting smaller objects^24,47^. Even for regions with a relatively high defect density (e.g., region D1 in Fig. 5b), the defect cluster location and size are correctly predicted. Because the defect clusters are small and require spatially accurate predictions to obtain high IoU values, our choice of the anchor-free detector (Fig. 3d) leads to better location and size predictions. This ability of *DefectTrack* in resolving defect clusters’ centers with high accuracy will later benefit the extraction of the re-ID feature for tracking. In addition, our implementation of image augmentations further increased the F1-score by 5.76% (Fig. S3c), as it works by expanding the range of feature possibilities such that the dataset can capture various corner and edge cases.

To analyze the source of detection errors, we first examined the missed detections (FN). As shown in Fig. 5a–e, the FN detections (yellow boxes) in the comparison maps suggest the errors are mostly related to defect clusters with considerably weak contrast. Compared to these weak-contrast defects, the defect clusters with sharp contrast (indicated by turquoise arrows) were detected with high confidence. However, during their lifetimes, most defect clusters exhibit significant modifications in size and contrast, due to growth and recovery and changes in local TEM diffraction conditions^48^. This dynamic evolution of defect clusters might lead to missed detections in some frames even though it is tracked for most frames (Fig. S5a). We then modified the pixel-wise logistic regression with designed focal loss (α = 3, β = 5) to handle these weak-contrast defect clusters^33^. The focal loss works by dynamically scaling the loss based on the detection confidence scores. It weighs the harder (low confidence) to detect defect clusters more than the easier (high confidence) ones and improves the overall detection performance. Although some defect clusters with uncommon feature representations were still missed, without generating additional training data (which is expensive), a 4.35% gain in recall was achieved through this loss function modification.

Lastly, we analyze the source of the false detections (FP), marked by red boxes in the comparison maps in Fig. 5a–e. We found that most FP predictions have a lifetime of one to two video frames. Some FP detections can be attributed to the background noise that causes random intensity fluctuations in TEM images that look resemble a white–black lobe pattern. Other FPs (e.g., marked by red arrows in Fig. 5a–c) are found to contain high-intensity pixels and are along the image edge boundaries (red arrows in Fig. 5d–f). Since the size of these defects is small, a slight fluctuation in the areal intensity can be confused as a defect cluster, and thus leads to FPs. Nevertheless, considering that *DefectTrack’s* standalone detector performance is already close to the SOTA interpolated detectors, we decided to halt further detector optimization, and treat the FP and the remaining FN errors in subsequent tracking training. Specifically, as Re-ID and visual tracking can access the detection information from past frames, we expect to use them to reduce detection errors. These further mitigation strategies are discussed in the following tracking section.

### Tracking of defect clusters

In this section, we focused on training the *DefectTrack* on tracking the defect clusters in the in-situ TEM video dataset. By using the model trained first on defect detection, we then jointly trained the detector and Re-ID branch. Collectively, our *DefectTrack* demonstrates a competitive tracking performance. Below, we first summarize the tracking performances and then discuss the steps taken to achieve this performance. Table 2 summarizes the averaged tracking performance over the eight test sets. An MT of 67.81 ± 2.07%, MOTA of 66.43 ± 2.32%, and IDF1 of 57.38 ± 1.81% were obtained. In the recent MOTChallenge, the SOTA MOT models GMOTv2 reported an MT of 72.1%, MOTA of 72.9 ± 13.4%, and IDF1 of 69.2 ± 12.1%, while TransCenter^46^ has MT of 48.4%, MOTA of 57.2 ± 22.2%, and IDF1 of 46.4 ± 14.8%^28^. When compared to *DefectTrack’s* tracking performance, it is evident that our model performance is competitive. Another key feature of a good tracker is model stability. In particular, the model performance is robust to training-test data partitioning. We found that the standard deviation of MOTA of *DefectTrack* is small (± 2.32%), when compared to GMOTv2 (± 13.4%) and TransCenter (± 22.2%)^28^. This suggests that our model is quite stable. While image contrast varies in different divided video sequences (Fig. S2) derived from one TEM video, we think this contrast variation is largely mitigated by the variance normalization applied. Thus, proper image pre-processing applied prior to model training is beneficial to enhancing model stability.

**Table 2:** *DefectTrack*’s multi-object tracking performance.

Model performance	MT ↑	MOTA ↑	IDF1 ↑	ID Sw. ↓	FPS ↑
Eight-fold cross validation	67.81 ± 2.07	66.43 ± 2.32	57.38 ± 1.84	89 ± 12	28

Average tracking performance and standard deviation on the eight test sets are presented here for eight-fold cross validation. Note that the up arrows indicate the higher the score the better the tracking performance, and vice versa. For details of the MOT metrics, see Supplementary Sect. 4.

**Figure 6:**
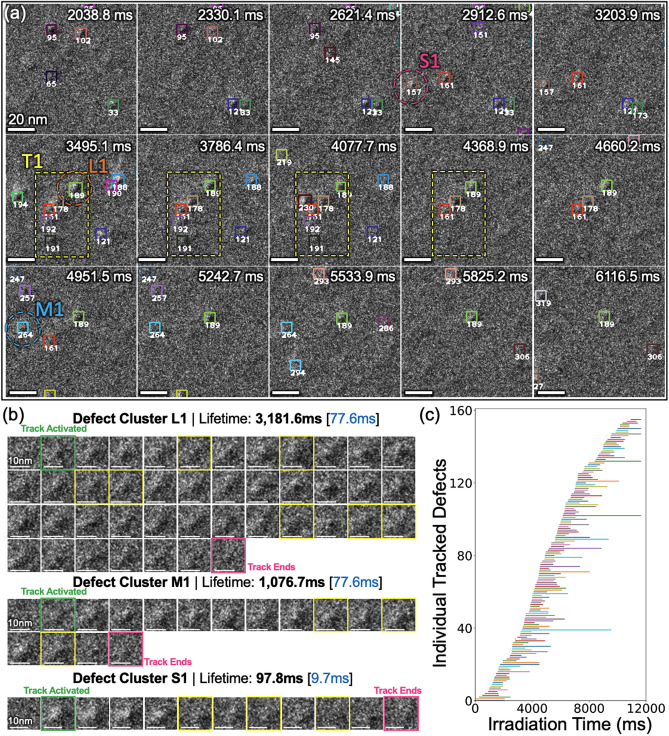
Visualization of the *DefectTrack’s* multi-object tracking performance on a representative test set. (**a**) Cropped 256 × 256 video frames from the *DefectTrack* prediction on the test set with a time interval of 291.3 ms (30 frames). The tracked defect clusters are marked with a bounding box and unique tracking IDs in the video frames. The bounding box color is encoded based on its tracking ID. (**b**) Representative example of individual defect clusters tracked by the *DefectTrack*. The time interval between frames is indicated by blue text inside the bracket. (**c**) *DefectTrack* predicted defect cluster tracks. The start of the bar indicates defect formation, the length is the lifetime, and the end of the horizontal bar marks the annihilation of defect clusters during in-situ irradiation. Note that only defect clusters with a lifetime greater than 600 ms are shown here for clarity.

Fig. 6a illustrates *DetectTrack*’s tracking performance on a representative 256 × 256 pixels section of the test set. The color of a bounding box remains unchanged as long as the defect cluster is tracked with the same ID (i.e., the defect cluster is accurately tracked). As shown in the prediction overview in Fig. 6a, most of the bounding boxes maintain the same color throughout the video. This suggests that *DefectTrack* can successfully track defect clusters with different defect representations and lifetimes at the individual defect level. Specifically, in Fig. 6a, short-lived (S1), medium-lived (M1), and long-lived (L1) defect clusters are highlighted to demonstrate that our model can consistently track defect clusters with different lifetimes. In Fig. 6b, time series prediction is shown for the three defects. These tracked defects can be better visualized in Fig. 6c. Each horizontal bar represents a defect cluster and only defect clusters having long lifetimes greater than 600 ms are visualized. In short, our *DefectTrack* successfully tracked defect clusters with lifetimes ranging from 19.4 ms (2 frames) to 6,411.7 ms (660 frames).

Moreover, *DefectTrack* achieved a high average IDF1 of 58.74% (GMOTv2 IDF1 = 69.2%, TransCenter IDF1 = 46.4%)^28^ and a low average ID switch (IDSw) of 89 (GMOTv2 average IDSw = 421, TransCenter average IDSw = 1123)^28^. This means our model can accurately maintain the same ID during defect cluster tracking. Only in limited cases, as demonstrated by low IDSw, wrong ID assignment occurs even though an average of 30,381 defects clusters are encountered per video sequence (Fig. S2b). As marked in Fig. 6b, the tracking is activated (green frames) immediately after the defect cluster is first detected. Then, the re-ID branch encodes the defect appearance and is updated every frame. However, due to sudden change in defect appearance and/or weak defect contrast, some defect clusters are missed (outlined with yellow boxes in Fig. 6b) by the detection branch, and thus neither a re-ID vector is generated, nor a bounding box is predicted. This change in appearance is particularly obvious for the defect cluster S1. For such a case, the visual tracking is then activated. With the addition of visual tracker on the original algorithm, a significant performance gain is achieved, e.g., MT (up 18.29%), MOTA (up 12.67%), and IDF1 (up 6.10%). Furthermore, the *DefectTrack* successfully handles defect cluster tracking in dense defect regions like the T1 yellow dashed box in Fig. 6a). To precisely track defects in such scenarios, extracting accurate re-ID features is of utmost importance. As ambiguous re-ID features lead to track fragmentation (low MT), IDSw, and premature defect track termination. For this reason, we adopted an anchor-free detector in the *DefectTrack* and the HRNet-w18 for high-resolution representations, leading to robust re-ID features and precise defect cluster position and size prediction.

One remaining issue in model performance is related to the false positives. These errors can adversely affect the defect cluster lifetime distribution measurement. In our case, most false-positive predictions are single-frame detections that do not form defect tracks. For multi-object tracking, it is more important to track defects that exist in multiple frames. To enable tracking of *long-lived* objects MOT algorithms apply a threshold on the number of frames an object (defect clusters in our case) should be detected before they are tracked^21,24,36^. To repeat, we alleviated the issues of FP defect clusters (Fig S5c) by setting the minimum track length (*t*_min_) to two frames and defect tracks by requiring at least one defect in the track to have a high confidence score (σ_h_) of 0.6. In sum, the design of two-level tracking module of *DefectTrack* works in tandem and mitigates challenges posed by false positives and missed detections, leading to excellent tracking performance.

### Comparing defect lifetime distribution between *DefectTrack* and human experts

Standard MOT metrics are fundamentally different compared to materials science evaluation metrics. While *DefectTrack* processed the entire dataset in 57.14 s on NVIDIA QUADRO RTX 6000 GPUs, it took the human experts an average of 5.25 h to track 1/10 defect clusters (Fig. S6). For practical application to the materials science domain, it is necessary to also assess how well the model output captures actual physical phenomena. In our case, defect lifetime distribution is a crucial measure to understand defect evolution under irradiation conditions^48^. Understanding the relationship between this distribution and MOT metrics could help guide and determine the end goal of model training. Below, we first compare *DefectTrack’s* performance in predicting the defect lifetime distribution with that of human experts using the Two-sample Kolmogorov–Smirnov (KS) Test^43^ and the Two-sample Chi-Square Test^44^, and then discuss the relationship between the evaluation metrics (machine learning vs. the material science domain) for defect cluster lifetime distribution prediction.

**Figure 7:**
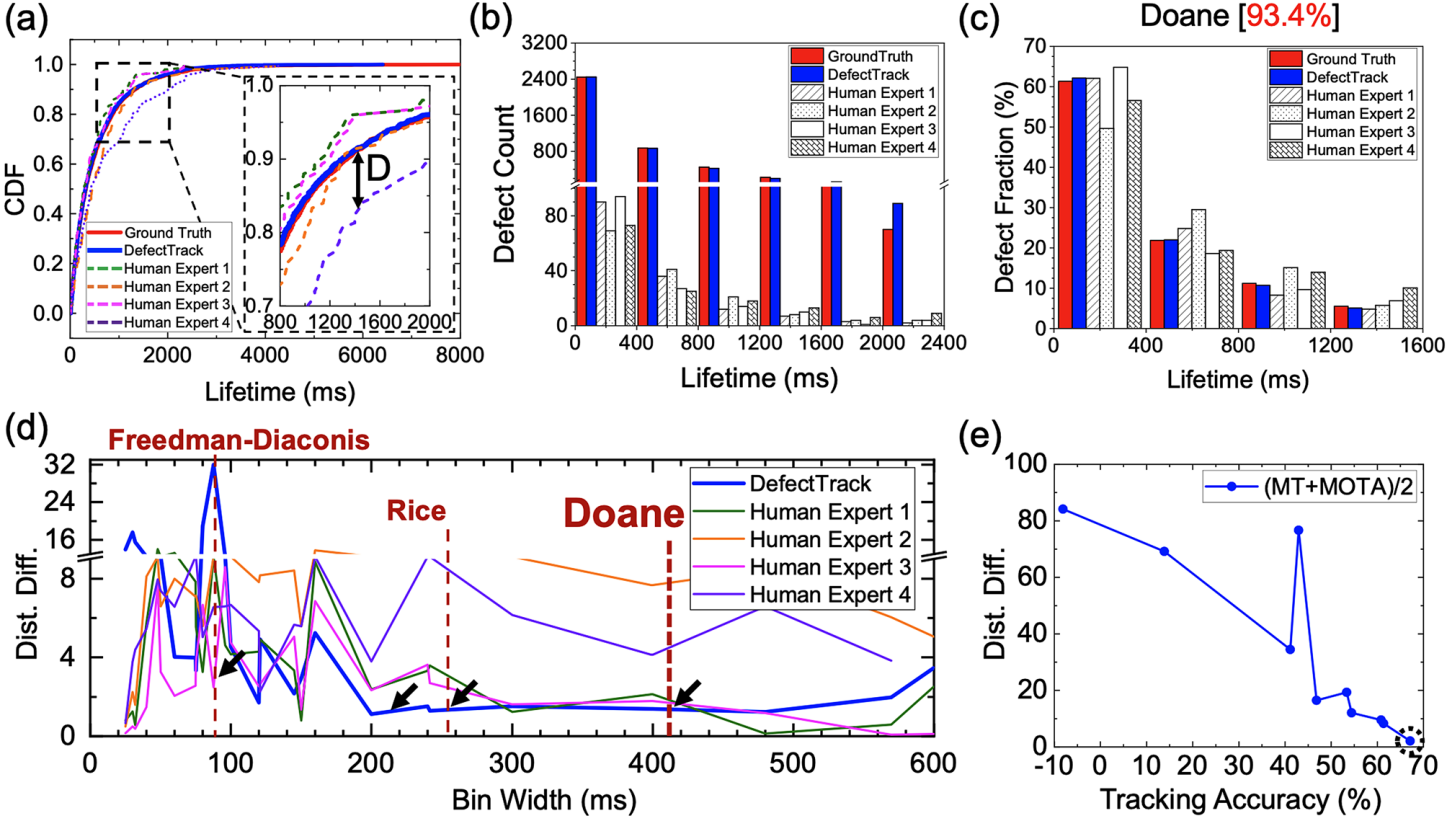
Comparison of the defect lifetime distribution prediction by *DefectTrack* and by Human Experts with the ground truth. (**a**) The cumulative distribution functions (CDF) of defect lifetime distribution of ground truth, as predicted by *DefectTrack,* and four human expert measurements. (**b**) Histogram showing the true lifetime distribution, *DefectTrack* prediction, and Human Expert measurements. (**c**) Normalized lifetime histogram. 93.4% of ground truth was included in the comparison after lifetime cutoffs. (**d**) The calculated distribution-difference (Dist. Diff.) for different bin widths (Fig. S7 and Table S2). The black arrow points to the bin widths at which *DefectTrack* has the lowest distribution-difference. Note the distribution-difference comparison can only be made for each bin width, and not across bin widths. (**e**) The correlation between the calculated distribution-difference (Dist. Diff.) on the y-axis and the MOT tracking performance evaluation on the x-axis.

In the two-sample KS test, we test the null hypothesis that the predicted defect lifetimes (whether by *DefectTrack* or human experts) and the ground truth defect lifetimes are drawn from the same distribution. Table 3 lists the KS test results for the defect lifetime distribution prediction made by the model with that of four human experts, including the KS test statistic (D) and corresponding p-value. The p-value is a statistical measure that estimates the probability of obtaining the observed test results assuming that the null hypothesis is true (details see Supplementary Sect. 5). We note that at significance level $\alpha$ = 0.05, we can reject the null hypothesis for humans 2 and 4 since the p-value is less than $\alpha$, and conclude that there is a statistically significant difference between the ground truth lifetime predictions and the predictions made by humans 2 and 4. In the case of *DefectTrack* and human experts 1 and 3, we cannot reject the null hypothesis. Note that although the test statistic *D* (0.018) is the smallest for *DefectTrack*, it does not have the highest p-value. This is because the large sampling size or total defect clusters tracked by *DefectTrack* (4378) requires a relatively lower *D* to achieve the same p-value as the human experts who only tracked 150 defect clusters^44^. While it is not in general appropriate to compare p-values, in this case, it is useful to note that *DefectTrack* achieves a higher p-value than human experts despite having a much larger sample size. For *DefectTrack,* the p-value is 0.488, which is much higher than human experts 1, 2, and 4; only human expert 3 has a higher p-value of 0.725. Considering that the much larger sample size of *DefectTrack* makes any differences between the empirical cumulative distribution functions (CDFs) more statistically significant, the lack of statistical significance observed by the KS test suggests that *DefectTrack*’s predictions match well with the ground truth. Fig. 7a presents the empirical CDF of defect lifetimes according to the ground truth, *DefectTrack*, and four human experts. It is clear that *DefectTrack* prediction closely follows the ground truth. We performed additional KS tests with smaller sample sizes by randomly selecting 150 defect clusters from the *DefectTrack* prediction with replacement (Table 3 and Fig. S7). The new p-values of 0.541 and 0.547 for two such random samples are consistent with the earlier result. In short, *DefectTrack* can predict the defect lifetime distribution much better than three out of the four human experts. Furthermore, the results in Fig. 7a show a wide variation among the lifetime distributions measured by human experts. This suggests that automated and robust techniques can remove subjectivity in the analysis of TEM images. To summarize, *DefectTrack* outperforms human experts at quantifying the defect lifetime distribution.

**Table 3:** Kolmogorov–Smirnov test results for comparing the defect cluster lifetime distribution predicted by *DefectTrack* and human experts measurements with the ground truth.

Prediction	Test Statistic (D)	P-Value ↑
*DefectTrack *(*N* = *4378*)	0.018	0.488
*DefectTrack-1 *(*N* = *150)*	0.065	0.541
*DefectTrack-2 *(*N* = *150)*	0.065	0.547
Human Expert 1 (*N* = *150)*	0.077	0.336
Human Expert 2 (*N* = *150*)	0.141	0.005
Human Expert 3 (*N* = *150*)	0.056	0.725
Human Expert 4 (*N* = *150*)	0.150	0.002

N is the sample size, i.e., the total number of tracked defect clusters. *DefectTrack-1* and *DefectTrack-2* are results of the Kolmogorov–Smirnov test performed by random sampling of 150 defect clusters from the total predicted by *DefectTrack*.

We also assessed the similarity between predicted and ground truth histograms, using the Two-sample Chi-Square Test (a binned test)^44^. This test is important because defect lifetime distribution data is commonly described by histograms^48^. First, we discuss the design of the test. In order to make a fair comparison and to accommodate: (i) the small number of defects tracked by human experts, and (ii) the shorter lifetimes of defects tracked by human experts 1 and 3 (with the longest lifetime of 2400 ms), we adopted the two-sample chi-square test for shape^44^ (i.e., make use of normalized histograms), and only compared the distribution of defect clusters with a lifetime of less than 2400 ms. Fig. 7b shows the raw defect lifetime count histogram with a lifetime of 0 to 2400 ms (Fig. S8a shows the entire lifetime histogram comparison). The number of defects predicted by *DefectTrack* and in the ground truth for each bin is 20 to 30 times higher than that of human experts. A common rule of thumb for the chi-square test is to have a minimum bin count of 5. To accommodate this, for a given histogram bin width we also applied a corresponding lifetime cut-off to remove bins with lower counts corresponding to longer lifetimes, so that all bins have a count of at least 5. To emphasize, tracking defects with a long lifetime is important because the statistical distribution of lifetime can be used to infer the nature of the defects such as interstitial dislocation loops, vacancy dislocation loops or stacking-fault tetrahedra as the stability of different defect clusters differs^48–50^. *DefectTrack* is capable of tracking defect clusters with a lifetime of up to 6411.7 ms or 661 frames (Fig. S8a). In contrast, manual tracking, which is inherently error-prone and laborious, human experts can only analyze a reasonable number of frames (~ 2400 ms; 97 frames). Long-lived defect clusters are likely missed in manual analysis, skewing the distribution to shorter lifetimes.

In this work, in addition to using the Chi-Square test statistic to determine statistical significance (p-value), we also use it to compare the substantive significance (effect size) of the results in our analysis. We use the term “distribution-difference” to refer to the Chi-Squared test statistic to emphasize that the comparison is being made under the same degrees of freedom, and in the equal sample size setting via normalized histograms so that the effect size varies monotonically with the test statistic (Details see Supplementary Sect. 5). Fig. 7c presents a normalized lifetime histogram with a cut-off at ~ 1600 ms. The bin width was determined using the Doane method^51^, and 93.4% of the ground truth data is included in the comparison. It is well known that the bin width used to perform the Chi-Square test can significantly affect the Chi-Square statistic (${\chi }^{2}$) value, and this is indeed observed in our calculated distribution-difference (Fig. 7d). Hence, we computed the distribution-difference for histograms with various bin width determination methods such as Doane^51^, Freedman-Diaconis (FD)^52^, Stone^53^, Scott^54^, and Rice^55^ to check this influence (Fig. S8b–e and Table S1 and S2). Additionally, we selected bin widths that are factors of 2400 ms. A low ${\chi }^{2}$ statistic corresponds to higher p-values. For the same bin width, a higher ${\chi }^{2}$ indicates that differences between the predicted and the ground truth distributions are more statistically significant. We found that the relative ranking of distribution-difference values between *DefectTrack* and the four human experts remains approximately stable for bin widths greater than 160 ms and less than 420 ms (Fig. 7d). In Fig. 7d, we use black arrows to mark the bin widths for which *DefectTrack* has the lowest distribution-difference. It is evident that *DefectTrack* has the lowest distribution-difference at many bin widths (i.e., by methods designed by Scott, and Rice). *DefectTrack* performs the best when applying the Doane method; the distribution-difference or ${\chi }^{2}$ is 1.379 (*df* = 3) which has a p-value of 0.71 (Table S1 and Table S2). In particular, Doane is the most appropriate binning method for this dataset since it was developed for non-normal and skewed data (exponential distribution has a skewness of 2^56^). For smaller bin widths produced by other methods such as FD, the lifetime cut-offs are necessarily very short to ensure a minimum count of 5 per bin, so the comparisons are likely invalid since a large proportion of defects are not considered. We have also assessed the lifetime distribution histogram by applying probability binning (Fig. S9 and Table S3), which allows for comparison using the entire ground truth data without a lifetime cut-off. The results further demonstrate that *DefectTrack* outperforms human experts in tracking defect clusters to estimate the actual lifetime distribution of defects.

Fig. 7e presents a comparison between the distribution-difference and the average of two important MOT metrics—MT and MOTA—which we collectively refer to in this section as tracking accuracy. Here, the distribution-difference refers to the Chi-Square test statistic calculated using the Two-sample Chi-Square test for shape with normalized histograms as described above and Doane-based bin widths, so that we can use their values for trend comparison even though the total defect count may be different at different confidence cut-off thresholds. We found that the higher the tracking accuracy, the lower the distribution-difference and the better the *DefectTrack* predicts the lifetime distribution. To enable this comparison, we gathered MT and MOTA values (Fig. S10a) and the distribution-difference for *DefectTrack* prediction at various confidence thresholds (Fig. S10b,c) and found that only having a high MT or a high MOTA is not sufficient to achieve a low distribution-difference. This is most likely due to the high numbers of false positives at high MT, which is obtained at low confidence thresholding, and similarly a high false negative rate at low MT due to a high confidence threshold (Fig. S10a,b). Although a higher MT can be achieved at lower tracking confidence thresholds, the MOTA is significantly worse, mainly due to the increase in false negatives (Fig. S10a). It leads to an error-prone estimation of the lifetime distribution. Similarly, simply looking at MOTA when assessing the tracking performance also leads to errors in lifetime measurements. A high MOTA score does not correspond to the lowest distribution-difference (Fig. S10c). Also, the error in lifetime distribution estimation increases at MOTA ~ 80% due to a large number of false negatives. Thirdly, it does not account for the number of defect clusters that are mostly tracked (MT). Lastly, there is an unexpected rise in distribution-difference at MOTA around 40%. This might be due to a combination of high false positives and false negatives, which affect the tracking capability of *DefectTrack.* Overall, high MOTA and MT scores are both required for a good prediction of the lifetime distribution.

## Conclusion and outlook

This work demonstrates the viability of *DefectTrack*—a deep learning-based one-shot MOT algorithm—to track dynamically evolving defect clusters in in-situ irradiation TEM video data for the first time. To enable this development, we established an in-situ TEM video ground truth for irradiation-induced defect clusters following the standard MOTChallenge protocol. The presence of weak-contrast defect clusters, due to their growth and recovery as well as changes in local TEM diffraction conditions, is one of the primary sources of uncertainties in MOT prediction. To overcome these challenges posed by the *real* in-situ TEM video data (not simulated ones), we found that the implementations of proper image pre-processing, a suitable MOT framework and loss function, and strategic optimizations are necessary for a successful MOT model. Moreover, we also found that training *DefectTrack* on defect cluster detection first and addressing the sources of errors improve detection performance (F1-score = 79.38 ± 3.33), and ultimately promote the overall tracking performance. Within a minute, our model has demonstrated the capability of tracking 4,378 unique defect clusters with lifetimes ranging from 19.4 to 6411.7 ms. The ability to track defects with a long lifetime allows us to infer the nature of the defects. As a result, our *DefectTrack* has achieved high MT, MOTA, and IDF1 scores of 67.81 ± 2.07%, 66.43 ± 2.32%, 57.38 ± 1.81%, respectively, and is capable of performing simultaneous detection and tracking.

To further assess *DefectTrack’s* performance under criteria relevant to the materials science domain, we compared the defect lifetime distribution produced by this MOT model and by human experts. The Two-sample Kolmogorov–Smirnov Test shows *DefectTrack* does not produce statistically significant differences from the ground truth lifetime distribution, while two out of four human experts did. This points to the reliability of machine learning methods such as *DefectTrack* compared to more variable human experts. Similarly, the Two-sample Chi-Square test fails to reject the null hypothesis that the lifetimes of defects predicated by *DefectTrack* are drawn from the same distribution as the ground truth even at high significance levels. A quantitative comparison of the effect size, which we derive from the Chi-Square test statistic and name “distribution-difference”, confirms that *DefectTrack* matches the ground truth lifetime distribution better than human experts. Finally, *DefectTrack* is capable of identifying > 10 times more defect clusters with > 2.5 times longer lifetimes than human experts. The achieved reliable, accurate, and high-throughput defect tracking and quantification for analyzing large in-situ video data (gigabytes-to-terabytes in a single irradiation experiment) is essential for understanding defect evolution and the overall material response under irradiation conditions. The video processing rate achieved in this work (at 28 FPS) can be further increased to go beyond 100 FPS with future work adding dedicated hardware like field-programmable gate arrays and model compression techniques. We conclude that dedicated deep learning-based MOT algorithms developed on practical in-situ TEM videos have demonstrated great potential in revolutionizing real-time defect analysis and in promoting a statistically meaningful understanding of the irradiation effects.

## Supplementary Information

Supplementary Information.

## Data Availability

The *DefectTrack* model, training video datasets, and ground truth labels of the current study are available in the repository (https://figshare.com/s/9e7f6c0870e828dbc1a2).
