# Supplementary material for: DefectTrack: a deep learning-based multi-object tracking algorithm for quantitative defect analysis of in-situ TEM videos in real-time
Source: Sci Rep. 2022 Sep 20;12:15705. doi: 10.1038/s41598-022-19697-1 (PMC9489724; doi:10.1038/s41598-022-19697-1)
Supplement: Supplementary file 1 — Supplementary Information. [file 41598_2022_19697_MOESM1_ESM.docx]

**Supplementary Information**

***DefectTrack*: A Deep learning-based Multi-Object Tracking Algorithm for Quantitative Defect Analysis of In-situ TEM Videos in Real-time**

Rajat Sainju^1^, Wei-Ying Chen^2^, Samuel Schaefer^1^, Qian Yang^3^, Caiwen Ding^3^, Meimei Li^2^ & Yuanyuan Zhu^1^*

^1.^ Department of Materials Science and Engineering, University of Connecticut, Storrs, CT 06269, USA

^2.^ Nuclear Science and Engineering Division, Argonne National Laboratory, Lemont, IL 60439, USA

^3.^ Department of Computer Science and Engineering, University of Connecticut, Storrs, CT 06269, USA

**1. Thermal Drift Correction and Image Normalization**

The thermal drift in the dataset was corrected using an in-house built cross-correlation-based MATLAB^1^ drift correction algorithm^1^ that utilizes the sub-pixel image registration as detailed in Guizar-Sicairos et. al.^2.^ In this method, each subsequent frame *N* is compared against the preceding frame *N-1* via cross-correlation (Figure S1a), and based on the cross-correlation coefficient, either of the frames *N* or *N-1* was then shifted in the *X* and *Y*-direction to compensate for the drift. A reference region (Figure S1b) containing dislocation features that persisted throughout the video was selected for drift correction. Figures S1c and S1d show the initial and final positions of the reference dislocations before and after the drift correction was applied. This measured drift correction was applied to the entire video (Figure S1e-S1f). Figure S1g showcases the magnitude of (thermal) drift correction applied in the spatial directions for each frame, which was found to be mainly in the *Y-*direction in this in-situ TEM video. Lastly, the contrast of the defect clusters was enhanced by applying background subtraction and full variance normalization^2^ to the drift-corrected video (Figure S1h).


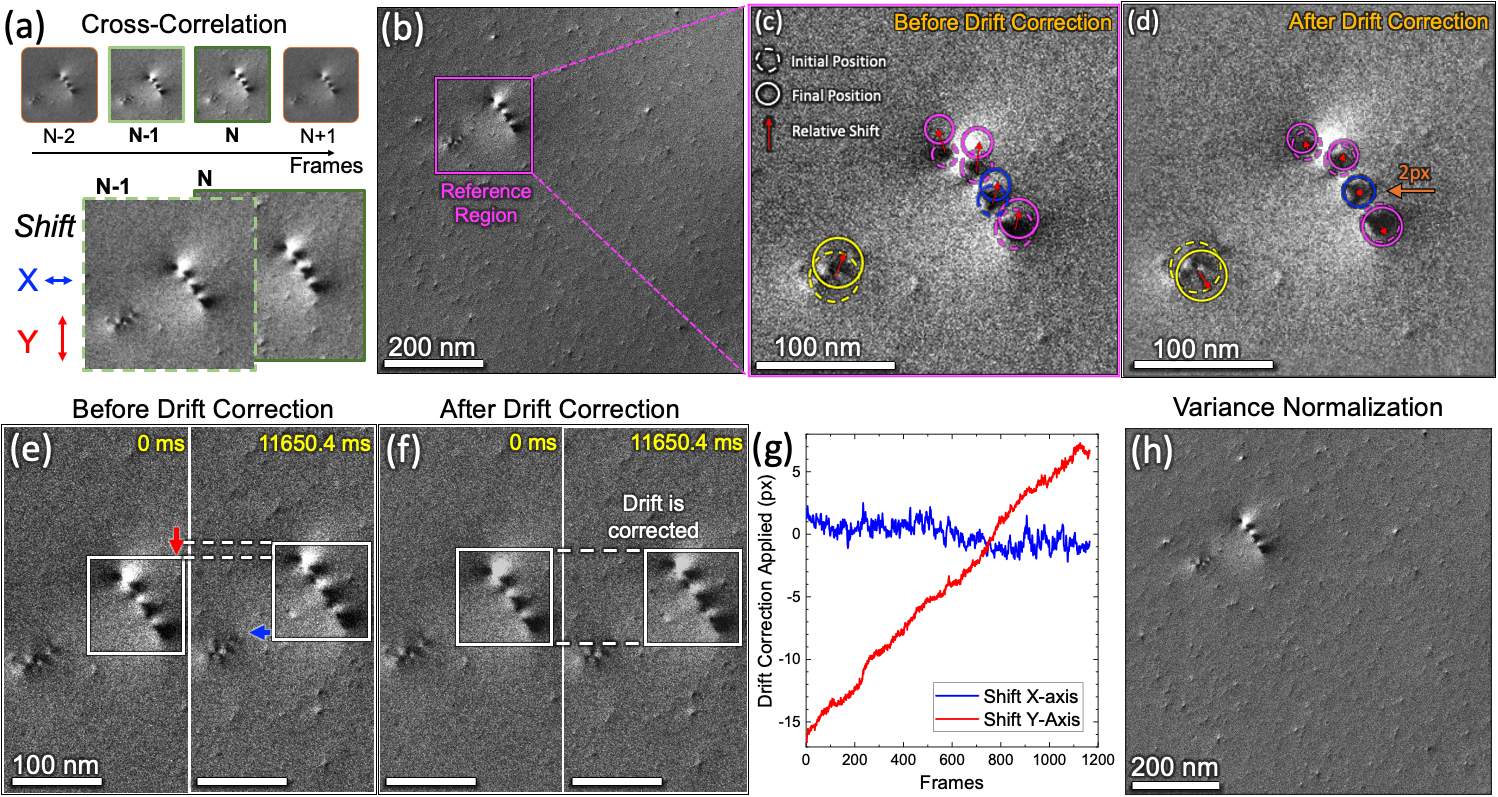


**Figure S1**. In-situ TEM video pre-processing including drift correction and image normalization. (a) Illustration of cross-correlation algorithm. (b) For better accuracy, a reference region is selected to correct the drift. (c) Illustrating the drift present in the whole sequence by comparing dislocation position at the first and the last frame. (d) Showing the same comparison as in S1c but in drift corrected images. The relative shift in dislocations is now pointing in different directions revealing real movements. This indicates that the linear drift is corrected. (e-f) Comparing the same region for drift correction in the first and last video frame (e) before and (f) after drift correction. (g) The total drift of ~25px is corrected in the *Y*-direction and ~3 px in the *X*-direction. (h) Background subtraction and variance normalization were applied to the image sequences to enhance the TEM image/defect contrast. The difference can be directly observed by comparing (b) with (h).

**2. Division of Annotated in-situ TEM Video for Model Training, Validation, and Testing**

Supplementary Figure S2a schematically shows how the ground truth labeled in-situ TEM video was divided into eight sequences. The total number of defect clusters and unique defect tracks is shown in Supplementary Fig. S2b.

**
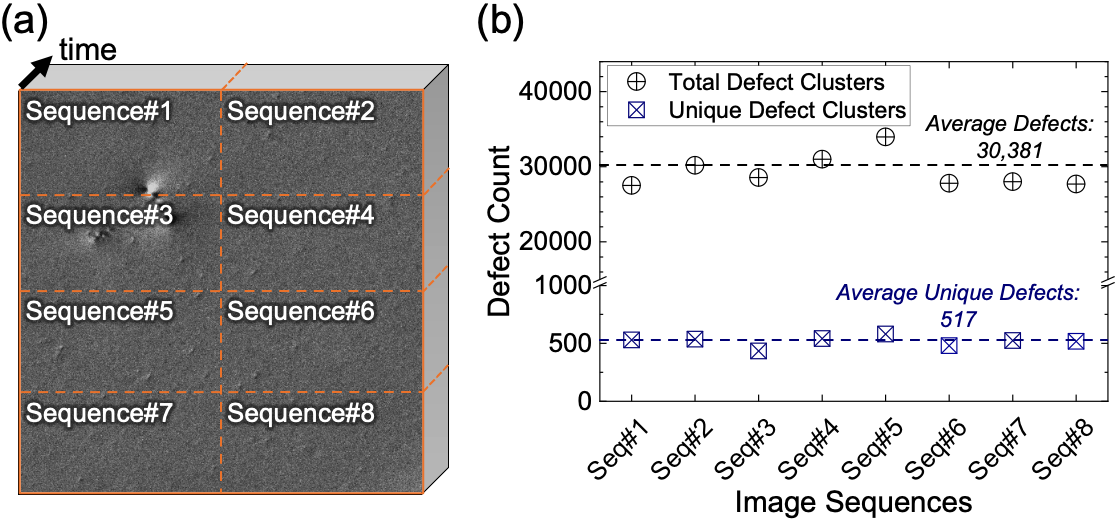
**

**Figure S2**. Schematics of the ground truth labeled in-situ TEM video divided into train-test sets. (a) The 1200-frame (each 2048 × 2048 pixels) TEM video was divided into eight smaller 1024 × 512 pixels frames of the same time sequences, which were used as for MOT model training, validation, and test. (b) The plot of the total numbers of defect clusters and the defect clusters with a unique ID in each of the eight divided sequences.

**3. Model Selection, Training Procedure, and Implementation Details.**

**Model Selection.** One-stage family of detectors balances the prediction accuracy and inference speed better than the comparatively slower two-stage frameworks^3^. Recent improvements applied to the one-stage family of detectors have even outperformed two-stage detectors^3^. We tested off-the-shelf backbone networks including HRNet-w18^4^, DLA-34^5^, ResNet-34^6^, and Yolov5s^7^ under the FairMOT framework, which have achieved remarkable performance in detecting everyday objects in real-time^8,9^. In our preliminary detection tests (Supplementary Fig. S3a), the HRNet-w18 demonstrated the highest detection accuracy measured using the F1-score. HRNet performs better in detecting small objects due to its demonstrated effectiveness in detecting relatively small objects in an image^9^, and is well-suited for our small defect clusters in the in-situ TEM videos. By connecting high-to-low resolution convolutions in parallel and performing multi-resolution fusions across scales, it creates semantically strong and spatially more precise features.^4^ Therefore, our *DefectTrack* is built on the FairMOT with the HRNet-w18 as the backbone network.


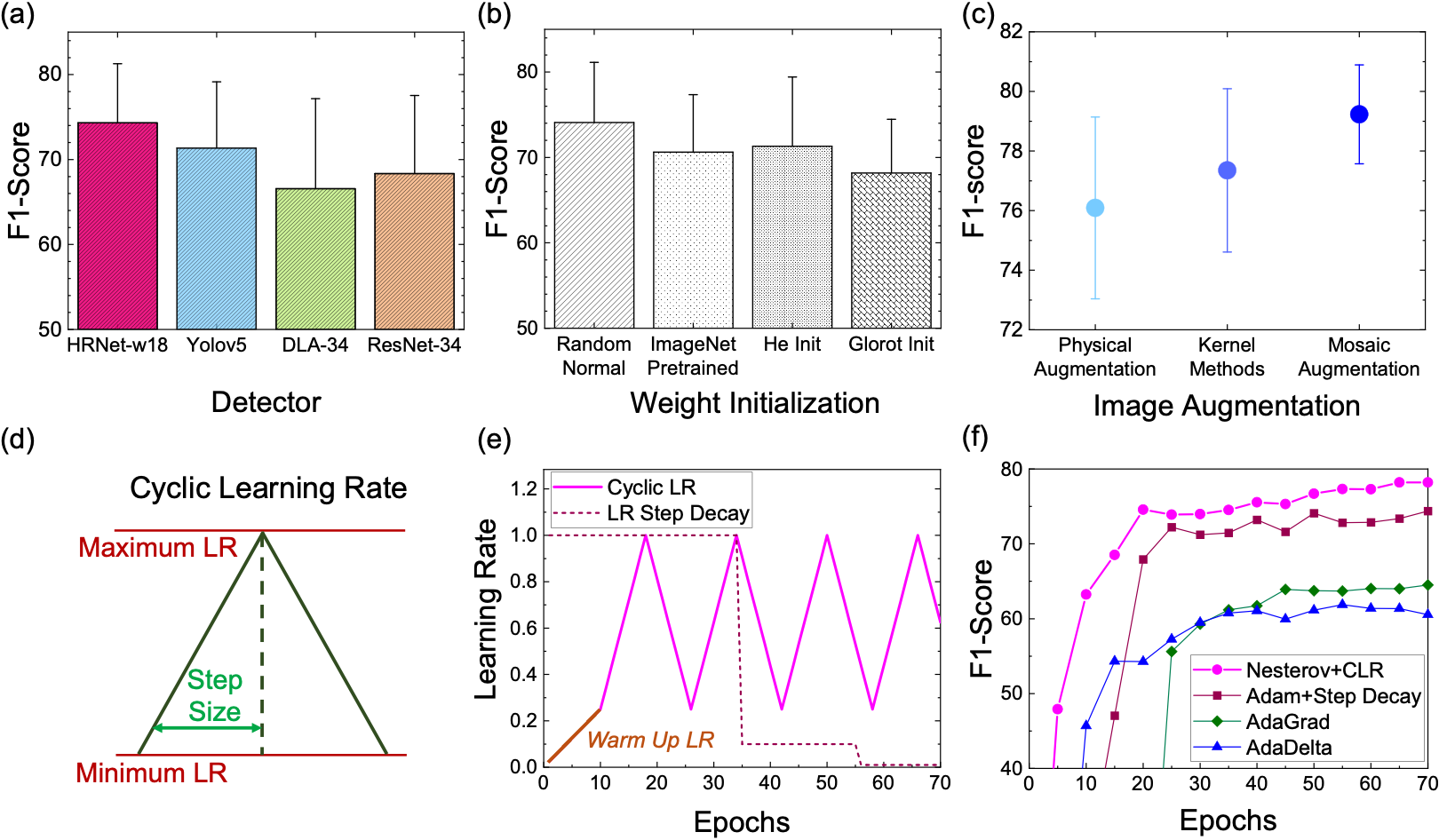


**Figure S3**. Initial convolution neural network (CNN) architecture selection and model training parameters. (a) Baseline detection F1-score comparison of four candidate models, including FairMOT with three backbone networks (HRNet-w18, DLA-34, and ResNet-34) and Yolov5s. (b) F1-scores of four tested model weight initialization methods. (c) Improvement in the F1 scores with each additional image augmentations method. (d) Diagrammatic representation of cyclic learning rate (CLR). (e) Comparison between the CLR and traditional learning rate step decay schedule. (f) Learning curves of test for model optimization comparisons.

**Training Procedure.** Different model training strategies were tested to achieve the highest detection performance on the dataset. F1-score was used to determine the best-performing training strategy. For successful application of deep neural networks (DNNs) proper strategy for weight initialization, data augmentation, optimization algorithm, regularization, and hyperparameters selection has to be applied. When training a neural network on a limited amount of electron microscopy data, applying transfer learning^10^ (e.g., using a pre-trained network on ImageNet) has been reported to improve the model performance^11^. However, we found that initializing the model weights using a random normal distribution with a standard deviation of 1E-2 results in the best performance (Supplementary Fig. S3b). This test was performed prior to the customization of the HRNet-w18 model. Two widely applied He initialization^12^ and Glorot initialization^13^ methods were also tested and compared. This observation suggests that using a model trained on everyday objects (e.g., the ImageNet) to detect the irradiation defects in electron micrographs does not necessarily lead to better model performance.

Image augmentation^10^ is another critical strategy to achieve strong generalization. In this work, we found that applying augmentations online during training outperforms using a pre-populated dataset (offline). Supplementary Figure S3c presents the incremental gain in the F1-score achieved by different augmentation strategies used in this work. We found that in addition to physical augmentation like rotation, flipping, translation, and scaling, applying kernel-based augmentation methods like a gaussian blur, median filter, random pixel drop, contrast stretching, Poisson, and additive Gaussian noise led to incremental performance gain. Furthermore, including mosaic augmentation^7^ provided additional performance gain by forcing the DNN to learn shape-related features rather than the simple contrast by creating a mosaic of training images from different time frames. . On the other hand, the effect of batch sizes on model training seems to be inconclusive in the literature. For instance, Goyal et al. commented that large batch sizes did not produce the best training results^14^. Group normalization and weight standardization suggested that small-batch training leads to higher accuracy^15^. Therefore, we carried out tests for relatively small batch sizes. However, small batch sizes only resulted in a slightly better detector performance of 0.4%, but the Re-ID feature learned was much worse. In general, there is no clear recommendation of the optimal batch size. For this reason, we then tested different batch sizes starting with the largest batch size that could be fit in our GPUs, and then randomly searched for the optimal batch size.

Learning rate is one of the most important hyperparameters. Cyclic learning rate (CLR)^16^, shown in Supplementary Fig. S3d, sets the learning rate to oscillate between the maximum and minimum bound after a specific step size (epochs) has been shown to train DNN much faster. In our training, we combined CLR with the Nesterov Accelerated Momentum^17^ the CLR performs better than the conventionally applied Adam optimizer^18^ with a decaying learning rate after 50% and 80% of training completion. Supplementary Figure S3e shows how the learning rate varies at different epochs. The Nesterov Momentum and CLR combination leads to higher performance and a faster model convergence than Adam and other optimizers with adaptive learning strategies such as AdaGrad^19^ and AdaDelta^20^ (Supplementary Fig. S3f). The effect of batch sizes on model training seems to be inconclusive in the literature (Goyal and Group Normalization weight standardization). While small batch sizes resulted in a slightly better detector performance of 0.4%, the Re-ID feature learned was much worse. For this reason, we then tested different batch sizes starting with the largest batch size that could be fit in our GPUs, and then randomly searched for the optimal batch size. In the initial model training stage, the initialized weights were random and far from the final values; thus, using a large learning rate early produces less informative gradients and leads to numerical instability. Therefore, at the initial stage of training, we set up a learning rate warm-up schedule^14^, where it is linearly scaled from 0 to the initial learning rate in m epochs. To prevent model overfitting, regularization techniques including weight decay, early stopping^10^, and monitor train-validation loss were applied. *DefectTrack* was trained on several hundreds of random hyperparameters. Furthermore, the best models and hyperparameter configurations were used to warm start additional training for performance improvement. The best models were selected based on the model performance on the validation set. Then, the best models were applied to the test set. To achieve optimal model configuration, and the model was selected based on the evaluation on the validation set using the *k*-fold (eight-fold in our case) cross-validation technique^21^. We report the test set performance and further evaluate the model stability^22^ among different combinations of the divided dataset.

**Implementation Details***. DefectTrack* was trained only for the detection during the first 70 epochs, and then the detection and re-ID branch were trained together for the final 30 epochs with loss functions as defined in^9^. We found that this strategy produces the best results as it resolves the learning conflict between detection and re-ID tasks. Similarly, the FairMOT used a detector previously trained on the MS-COCO^23^ dataset and trained the tracker only for 30 epochs^9^. The model was trained with a CLR varied between 1.9E-5 and 0.4E-5 for 100 epochs. A learning rate warm-up was applied for 10 epochs with a batch size of 24. The input image and output feature map sizes are 1,024×512 pixels and 256×128 pixels, respectively. *DefectTrack* was implemented using PyTorch^24^ and Python libraries. Training on one hyperparameter configuration takes 28 hours to complete on two NVIDIA QUADRO RTX 6000 GPUs.

**4**. **Machine Learning** **Detection and MOT Performance Assessment Metrics**

The machine learning performance evaluation metrics for detection and MOT, and statistical tests used to assess the defect cluster lifetime distribution predicted are described below.

$$\boldsymbol{Precision}= \frac{TP}{TP+FP} ; \boldsymbol{Recall}= \frac{TP}{TP+FN}; \boldsymbol{F}\mathbf{1}\boldsymbol{score}=\frac{2*Precision*Recall}{Precision+Recall}$$

$$\boldsymbol{AP}= \int_{0}^{1} P\left( R \right)dR$$

To calculate the above metrics, we firstly determined true positive (TP), false positive (FP), and false negative (FN) for object detection. Whether an object prediction is TP or FP, or FN depends on the value of Intersection over the Union (IoU). IoU is defined as the ratio of the overlapping area between the predicted and the ground truth area to that of the total area. An object prediction is categorized as a TP if its IoU is greater than a certain threshold; otherwise, it is considered an FP. We consider IoU of 0.3 as the threshold for TP detection (Supplementary Fig. S4). This choice is further discussed in main text **Results and Discussion > Detection of defect clusters**. AP is then calculated at the IoU threshold of 0.3 for a single class defined in the PASCAL VOC^25^.


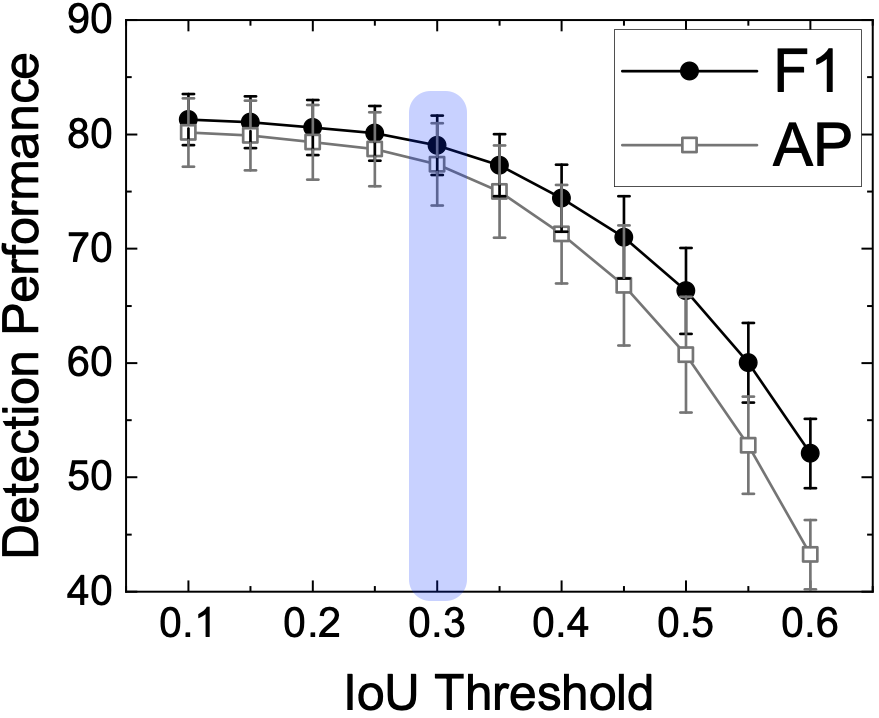


**Figure S4**. Choice of IoU threshold for *DefectTrack’s* detection performance evaluation.

To specifically assess the tracking performance, we used evaluation metrics including Mostly Tracked (MT), Multi-Object Tracking Accuracy (MOTA), IDF1, ID switches (IDSw). MT, MOTA, and IDF1 are defined below.

*MT* (Mostly Tracked) is defined as the percentage of objects tracked for at least 80% of the object’s lifetime^26^. It measures tracking quality, and a higher MT value means better tracking. An illustration of two cases that qualify as MT is illustrated in Supplementary Fig. S5a. The object does not need to be tracked continuously for 80% of its lifetime.

*MOTA* (Multi-Object Tracking Accuracy) is the most widely used metric to assess multi-object tracking performance^26^. It is also the benchmark MOT metric and is defined as:


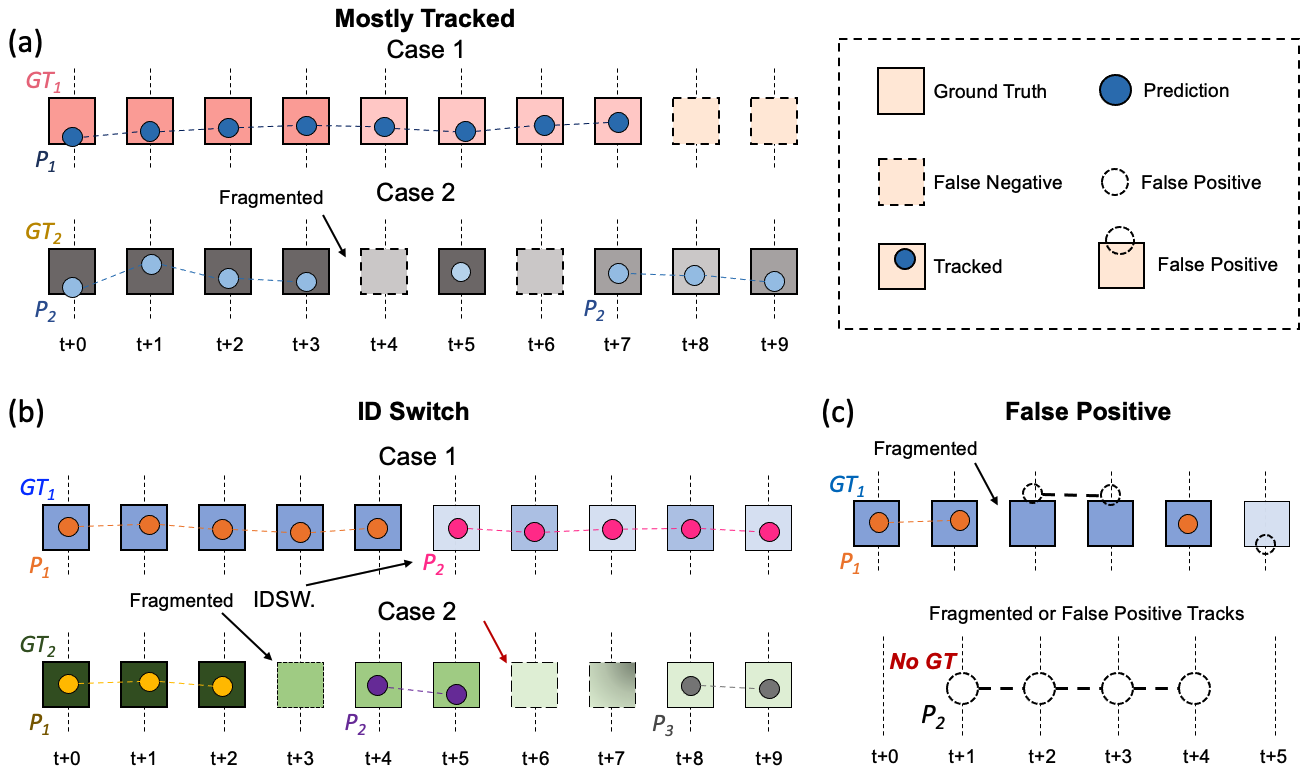


**Figure S5**. A visual explanation of the concepts of Mostly Tracked, ID Switches, and False Positive tracked. (a) Mostly tracked (MT) is a track quality measure which that evaluates whether an object is tracked for 80% of its lifetime. As Shown in Case 1, a defect cluster is tracked continuously for 8 out of the 10 frames or 80% of the lifetime. Case 2 presents two fragmentations but is still considered as MT. (b) Identity switch occurs when the same defect cluster is identified as a newly formed defect cluster and the model assigns it a different unique ID. Sudden appearance change (Case 1), track fragmentation, and missed detection or false negatives (Case 2) lead to ID switches. (c) False positive tracks are encountered when the detector makes false positive predictions for successive frames. It usually results in a *false* increase in the number of defect clusters tracked with a shorter lifetime (0-400ms).

$$MOTA=1- \frac{\sum_{t} (FN(t)+FP(t)+IDSW(t))}{\sum_{t} GT(t)}$$

where it is frame number, and GT(t) is the total number of ground truth objects in the frame. IDSW occurs when the same ground truth object is tracked with a different tracking ID in a later frame. Supplementary Fig. S5b illustrates the cases where GT_1_ is tracked with two IDs and GT_2_ is tracked with three. In both cases, the tracker fails to maintain one ID throughout the lifetime. The limit of MOTA is (-inf, 100], and a negative score is possible in the case of a large number of errors. Although MOTA is used as a score to rank state-of-the-art MOT models, it is debatable whether a single metric can genuinely assess the tracking performance.

*IDF1* is the ratio of correctly identified detections over the average number of ground-truth and computed detections^27^. False-positive tracks are illustrated in Supplementary Fig. S5c introduce error in the overall measurement of defect lifetime. Thus, we use MT, MOTA, and IDF1 for model performance assessment.

**5. Defect Cluster Lifetime Distribution Assessment using Statistical Hypothesis Tests.**

To compare the ground truth defect cluster lifetime distribution prediction performance of *DefectTrack* with that of the Human Experts we applied the Two-sample Kolmogorov-Smirnov Test^28^ and the Two-Sample Chi-Square Test^29^. The tests are described in brief below.

**Two-sample Kolmogorov-Smirnov (KS) test**^28^ is a statistical test that compares the empirical distribution functions of two samples under the null hypothesis that the two samples are drawn from the same distribution. It is based on the empirical cumulative distribution function (CDF) and does not assume any particular underlying distribution. The CDF of a random variable X, defined by $F\left( x \right)=P\left( X\leq x \right)$, is monotonically increasing and bound between 0 and 1. The empirical CDF is computed from a given sample. The KS test statistic (D) is the absolute maximum distance (supremum) between the ground truth and the measured CDF. Then, D is used to calculate the p-value, which provides information on the probability of obtaining a test statistic as extreme or more extreme than that calculated by the statistical test on the given sampled data if the null hypothesis is correct. Smaller p-value implies the difference between the two distributions is statistically significant. Typically, a statistical significance level of $\alpha=0.05$ is applied, where a result is considered to be statistically significant if p $\leq\alpha$, suggesting a rejection of the null hypothesis.

**Two-Sample Chi-Square test**^29^ is used to test whether two samples of data are drawn from the same distribution (null hypothesis). It is a binned test that compares the histograms of two distributions. A chi-square test statistic ($\chi^{2}$) is calculated and compared with a critical value (C_v_) at a specified significance level $\alpha$. The C_v_ is determined by the degrees of freedom $df$ (*df* = b – 1, where b is the total number of bins) and $\alpha$ (commonly use $\alpha$= 0.05). A higher $\chi^{2}$ value than C_v_ suggests that the null hypothesis should be rejected and the two sample distributions tested are likely to be different. In this work, since the distributions of defect cluster lifetime are described by the lifetime histograms, we applied the two-sample chi-square test for shape^29^ to assess to what extent the measured lifetime distributions (by *DefectTrack* or our Human Experts) are similar to the ground truth lifetime distributions. Note that the results of the Chi-Square test depend on the choice of the bin width, and it requires the same bin width and bin edges for both distributions for comparison. In this work, we are interested in comparing the performance of *DefectTrack* with human experts. Thus, in addition to studying whether or not there are statistically significant differences between the ground truth lifetime distributions and the *DefectTrack* or human expert lifetime distributions, we are also interested in comparing the effect size^30^ of these differences. Since measures of effect size for the chi-squared test such as Phi and Cramer’s V^31^ vary monotonically with the chi-squared test statistic for fixed sample size, we also use the chi-squared test statistic as a quantitative measurement for comparing the performance of predictions from *DefectTrack* or human experts. This is possible because we are using the two-sample chi-squared test for shape which normalizes the histograms to equivalent sample sizes. Thus in this work whenever we use the chi-squared test statistic to compare the substantive significance (effect size) of results in our analysis rather than just to measure the statistical significance (p-value), we use the term “distribution-difference” (Dist. Diff.) to refer to the chi-squared test statistic to emphasize that the comparison is being made in the equal sample size setting, and also under the same degrees of freedom.


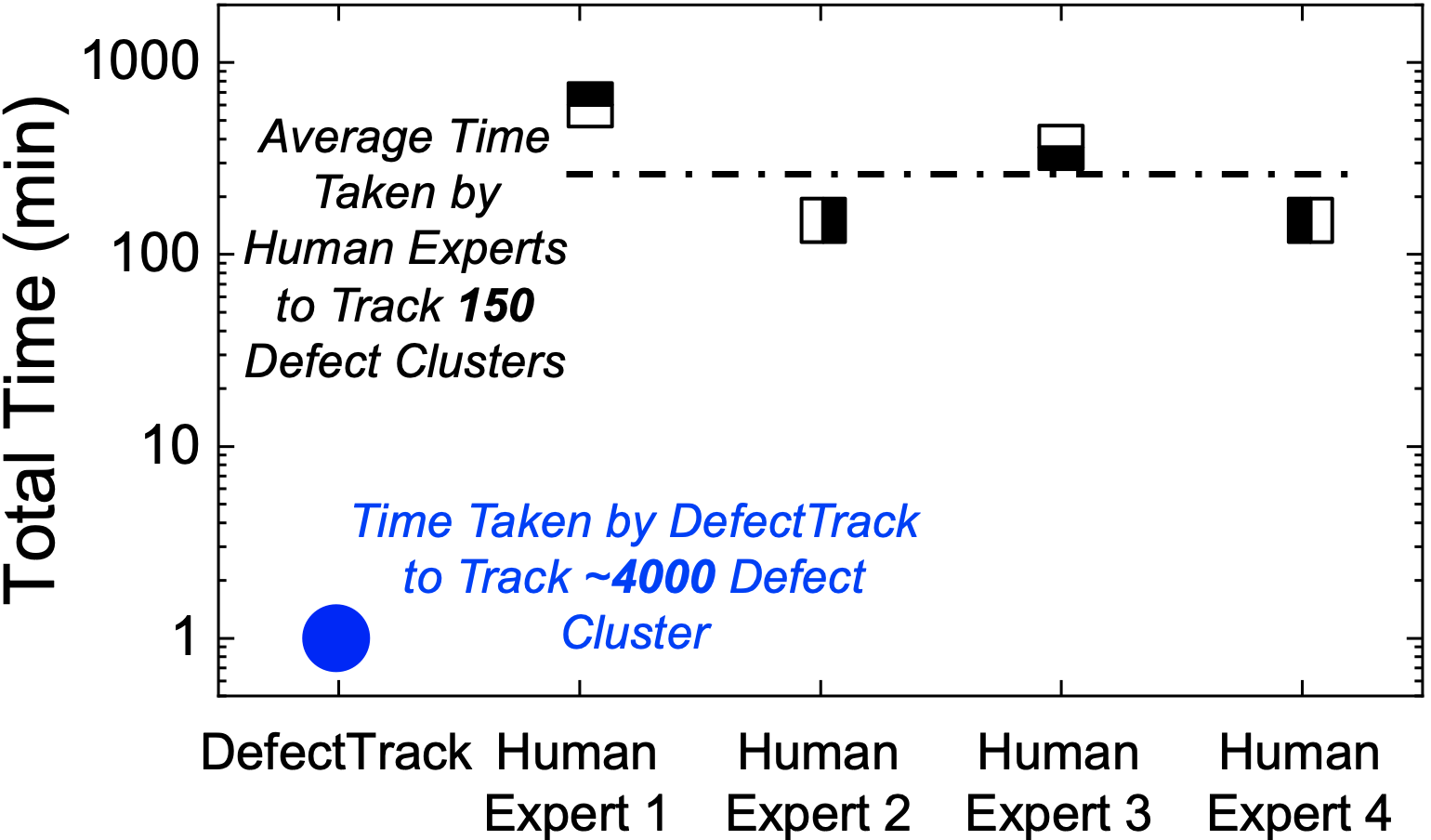


**Figure S6**. Comparing the time taken by *DefectTrack* to track more than 4,378 defect clusters with the time taken by human experts to measure 150 defect clusters. DefectTrack is able to analyze the *in-situ* TEM images at greater than 28 frames per second.

**
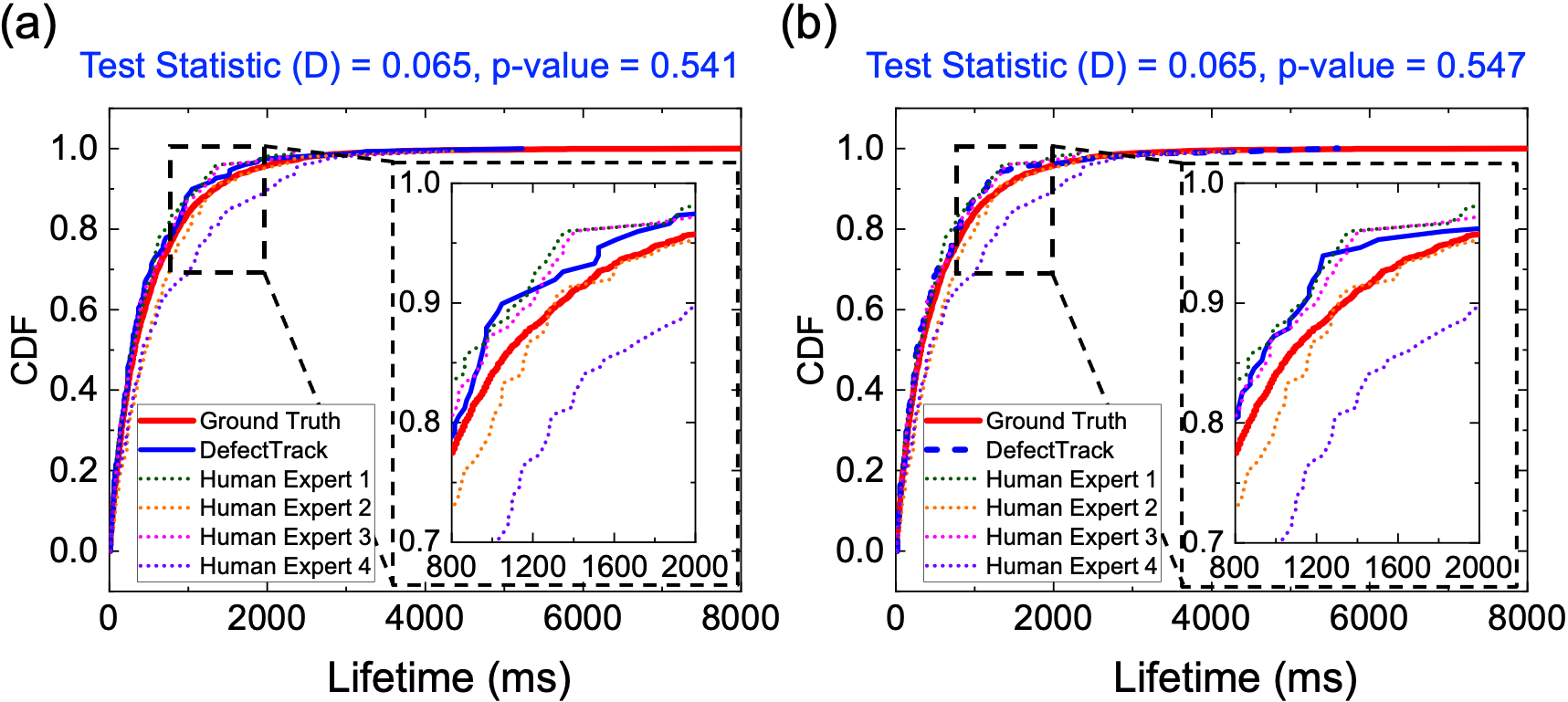
**

**Figure S7**. Downsampling of 150 defect clusters from the 4,378 defect clusters predicted by *DefectTrack* for Kolmogorov-Smirnov (KS) Test. The downsampling was performed by sampling uniformly at random with replacement. Compared to the main text Figure 7a, reducing the sample size to 150 now allows for comparison of the test statistic (D) with respect to the same sample size. Here (a) and (b) show the KS test statistic (D) and P-value obtained for two different random samples of 150 defect clusters.


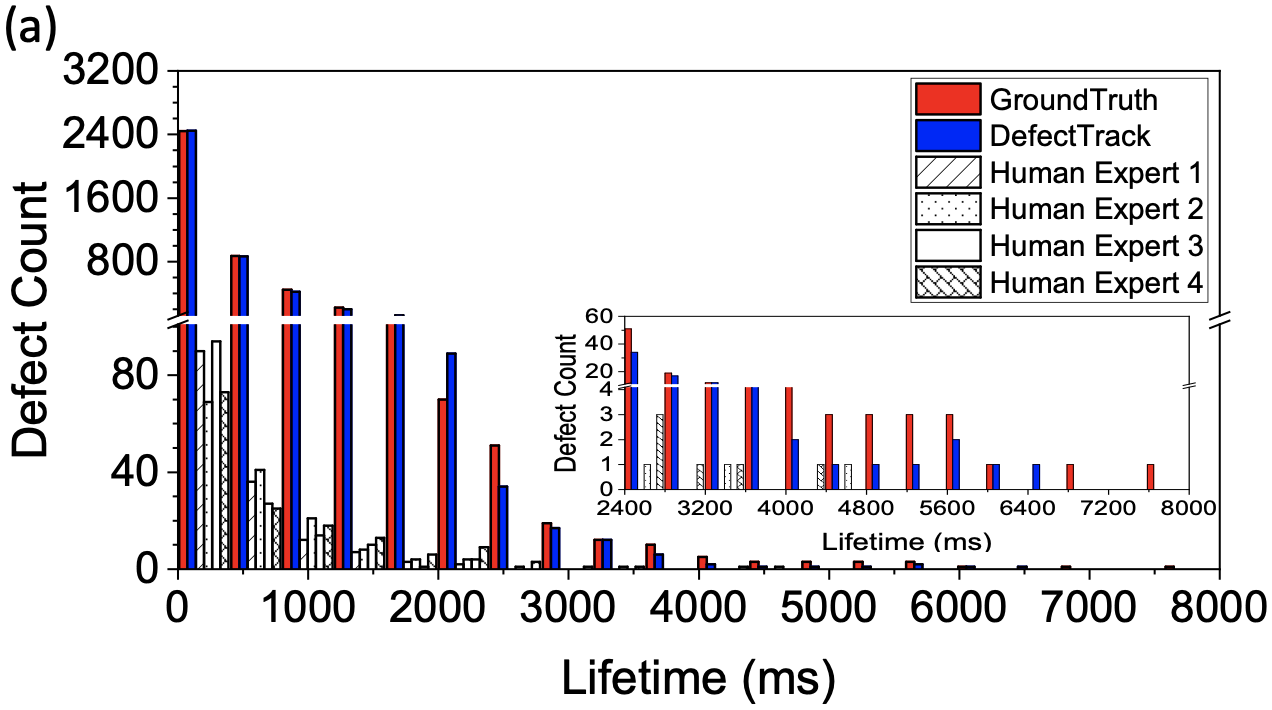


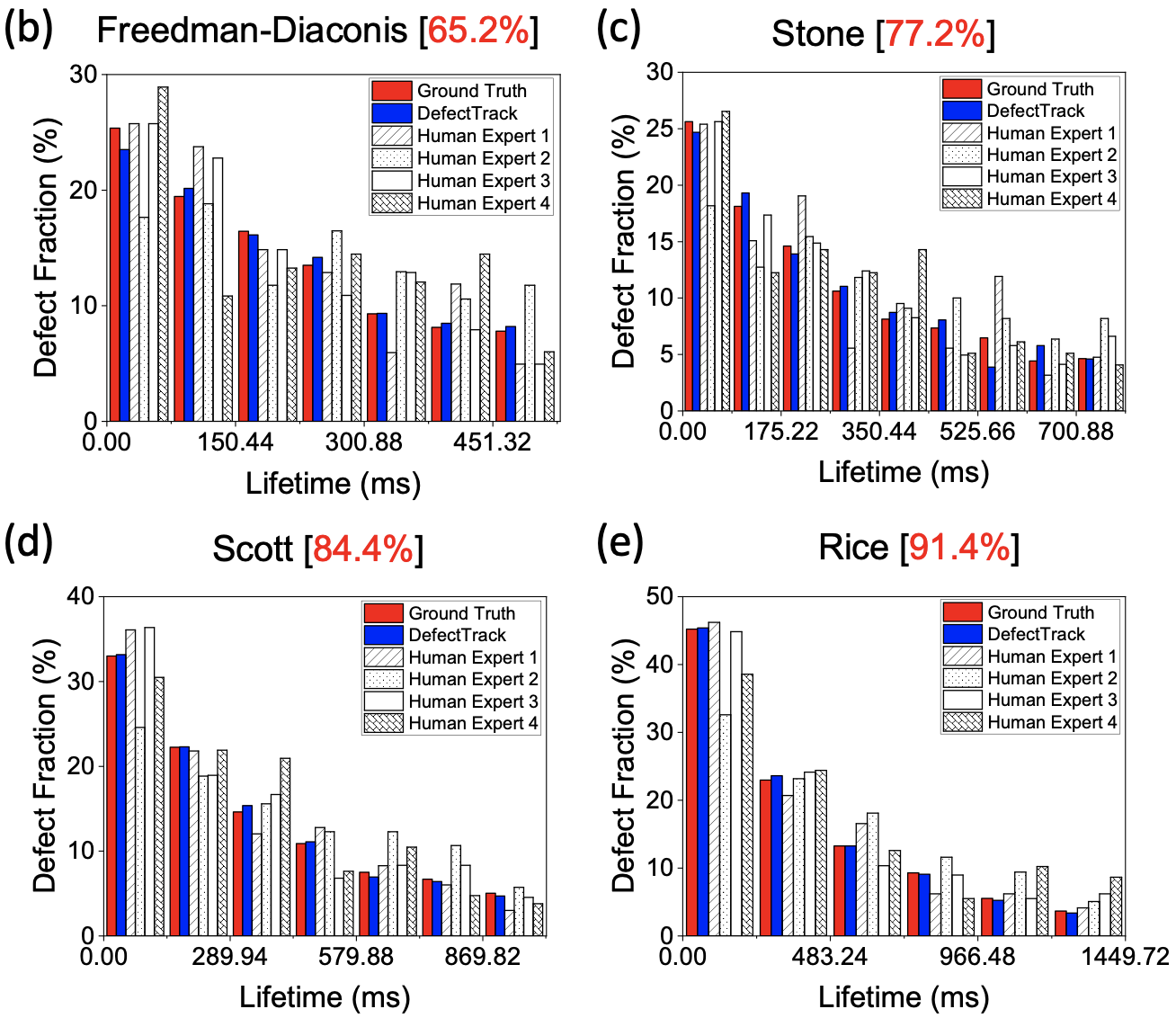


**Figure S8**. Defect lifetime histograms and normalized defect histogram with lifetime cut-offs. (a) Defect lifetime histogram without lifetime cut-off showing the lifetime distribution of defects in the ground truth, *DefectTrack* prediction, and human expert measurement. The bin width was determined using the Doane method. Defect fraction histograms with bin width determined by (b) Freedman-Diaconis, (c) Stone, (d) Scott, and (e) Rice method. These comparisons demonstrate the dependence of the resulting distribution on the bin width selection method. Note the lifetime cut-off is necessarily different for different bin size determination methods in order to maintain a minimum defect count of 5 in each bin. The percent of ground truth data included in the comparison is displayed in red in the figure titles.

| **Critical Values (C_v_)** | | Degree of Freedom (*df*) = ***Number of Bins - 1*** | | | | | | | |
| --- | --- | --- | --- | --- | --- | --- | --- | --- | --- |
|  |  | 2 | 3 | 4 | 5 | 6 | 7 | 8 | 9 |
| Significance Level | 0.50 | 1.386 | 2.365 | 3.356 | 4.351 | 5.348 | 6.346 | 7.334 | 8.343 |
|  | 0.25 | 2.770 | 4.110 | 5.390 | 6.630 | 7.840 | 9.040 | 10.22 | 11.39 |
|  | 0.10 | 4.605 | 6.251 | 7.779 | 9.24 | 10.64 | 12.02 | 13.36 | 14.68 |
|  | 0.05 | 5.991 | 7.814 | 9.487 | 11.07 | 12.59 | 14.07 | 15.51 | 16.92 |
|  | 0.01 | 9.210 | 11.344 | 13.278 | 15.086 | 16.811 | 18.48 | 20.09 | 21.67 |

**Table S2**. The critical values of the chi-square distribution at different significance levels and degrees of freedom.

| **Distribution -difference** | **Bin Width Determination Method** | | | | | | |
| --- | --- | --- | --- | --- | --- | --- | --- |
|  | FD | Stone | Sqrt | Scott | Rice | **Doane** | Sturges |
| **Bin Width** | *75.22ms* | 87.61ms | 120.81ms | 144.97 | 241.62ms | ***398.67ms*** | 569.52ms |
| **Lifetime Cut-off** | *526.54ms* | 788.49ms | 845.67ms | 1014.79ms | 1449.72ms | ***1594.68ms*** | 1708.56ms |
| **Num. of Bins (b)** | *7* | 9 | 7 | 7 | 6 | ***4*** | 3 |
| **Number of Defects** | *2792* | 3305 | 3395 | 3610 | 3910 | ***3985*** | 4024 |
| **Percent of GT Data** | *65.2* | 77.2 | 79.3 | 84.4 | 91.4 | ***93.1*** | 94.0 |
| **Degrees of Freedom (*df*)** | *6* | 8 | 6 | 6 | 5 | ***3*** | 2 |
| **DefectTrack** | *3.348* | 31.999 | 4.912 | 2.164 | 1.286 | ***1.379*** | 1.978 |
| **Human Expert 1** | *5.541* | 12.016 | 4.921 | 3.344 | 3.587 | ***2.121*** | 0.587 |
| **Human Expert 2** | *7.148* | 9.331 | 8.158 | 8.405 | 11.444 | ***7.681*** | 6.038 |
| **Human Expert 3** | *3.801* | 2.491 | 2.632 | 5.058 | 2.706 | ***1.794*** | 0.083 |
| **Human Expert 4** | *9.586* | 6.551 | 2.260 | 5.662 | 10.963 | ***4.142*** | 3.827 |

**Table S1**. Calculated distribution-difference (derived from chi-square test statistic ($\chi^{2}$) as described in the text) for histogram lifetime comparisons with constant bin width. The chi-square test was performed for *DefectTrack* predictions and Human Experts measurements by calculating the bin width with different bin width determination methods. Note that the **comparison being made here is between rows** for the calculated chi-square values between *DefectTrack* and Human Experts for a particular bin width determination method.

**Two Sample Chi-square Test Using Probability Binning**

By applying probability binning^28^, we compare histograms that have unequal bin widths (increasing bin widths). The main requirement for probability binning is that the bins for the ground truth dataset should have an equal count. Here, the number of bins for the histogram was selected such that the standard deviation between the bin counts had the lowest value and hence approximately equal counts. Then, the two-sample chi-square test is performed. Unequal bin widths allows a higher defect count in each bin and removes the need for a lifetime cut-off to ensure a minimum count per bin. The results are shown in Table S3.


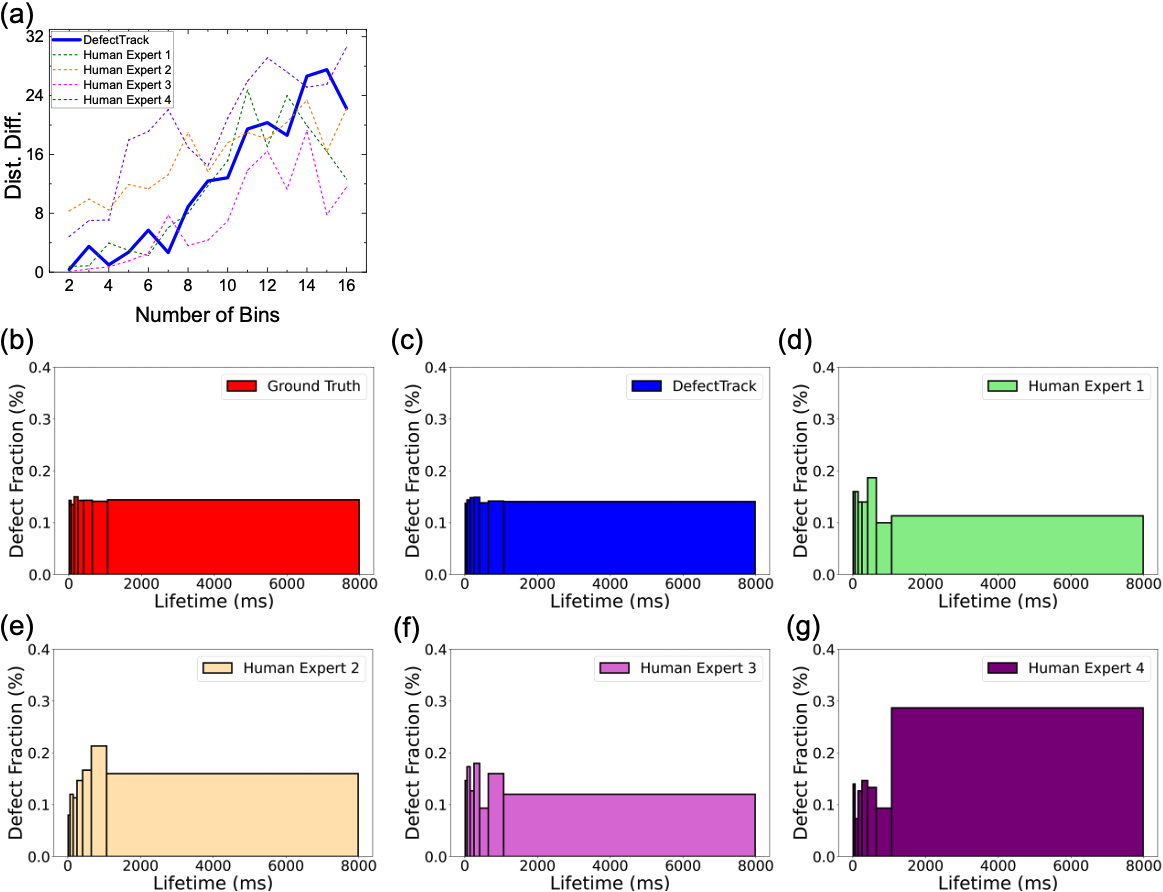


**Figure S9**. Comparing the *DefectTrack* predicted defect lifetime distribution with Human Experts measured distribution by applying probability binning. (a) Comparing the distribution-difference (Dist. Diff.) calculated using probability binning for DefectTrack prediction and human expert measurements. The value of the chi-square test statistic is dependent on the number of bins. (b) The resulting defect lifetime distribution of ground truth, (c) *DefectTrack*, (d-g) Human Experts, produced using the same bin edges with a total of 7 bins. The bin edges are chosen so that the ground truth bins have approximately equal counts with unequal and increasing bin widths.

**Table S3**. The chi-square value calculated for histograms produced by probability binning with different number of bins for *DefectTrack* and Human Experts. The critical values for different significance levels are also included in the table. Note the chi-square value for *DefectTrack* is not statistically significant at the $\alpha$ = 0.05 level from 2 up to 10 bins, which is not the case for all human experts. For larger numbers of bins, the chi-square value remains not significant at the $\alpha$ = 0.01 level, which is not always the case for human experts.

**
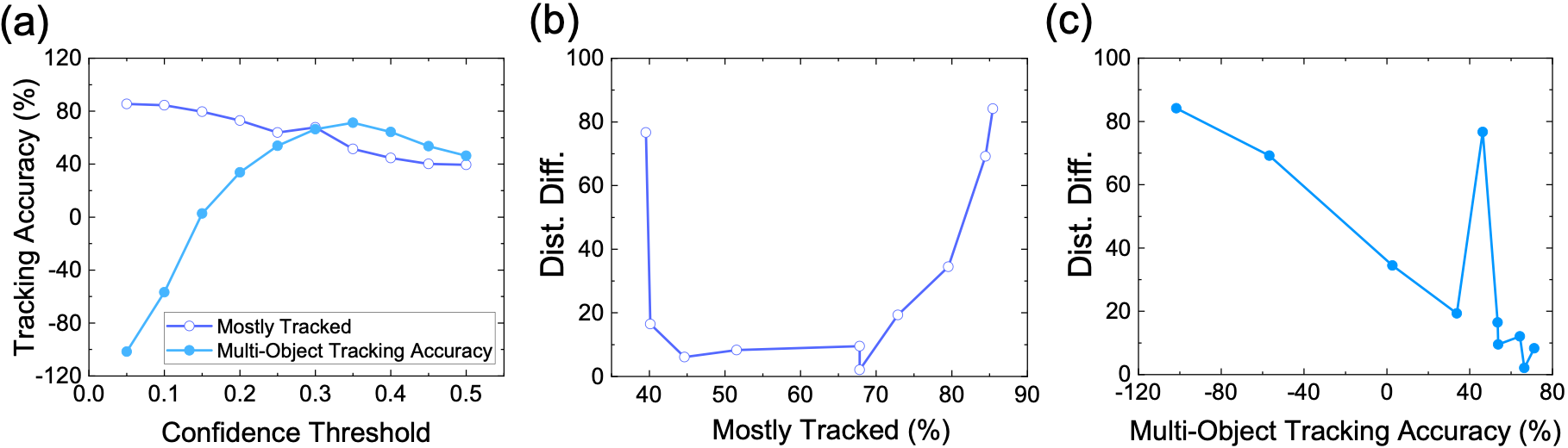
**

**Figure S10**. Correlation between domain-based statistical defect lifetime histogram evaluation and standard multi-object tracking performance. (a) *DefectTrack*’s tracking accuracy at different confidence cut-off thresholds. (b) The correlation between distribution-difference (Dist. Diff.) (domain-based defect lifetime histogram evaluation) and MT (standard computer-vision MOT performance evaluation metric). (c) The correlation between distribution-difference (domain-based defect lifetime histogram evaluation) and MOTA (a standard computer-vision MOT performance evaluation metric). Here the distribution-differences refers to the chi-square statistic calculated using Doane’s method for histogram bin-widths and the two-sample chi-squared test for shape. For more information on calculating the confidence threshold, please refer to the main text **Methods > Multi-Object Tracking** section.

**References**

1. MATLAB and Statistics Toolbox Release 2021a, The Mathworks, Inc., Natick, Massachusetts, U. S. MATLAB - MathWorks. www.mathworks.com/products/matlab.html (2021) doi:2016-11-26.

2. Guizar-Sicairos, M., Thurman, S. T. & Fienup, J. R. Efficient subpixel image registration algorithms. *Opt. Lett.* (2008) doi:10.1364/ol.33.000156.

3. Liu, L. *et al.* Deep Learning for Generic Object Detection : A Survey. (2018).

4. Sun, K., Xiao, B., Liu, D. & Wang, J. Deep high-resolution representation learning for human pose estimation. *Proc. IEEE Comput. Soc. Conf. Comput. Vis. Pattern Recognit.* **2019**-**June**, 5686–5696 (2019).

5. Yu, F., Wang, D., Shelhamer, E. & Darrell, T. Deep Layer Aggregation. *Proc. IEEE Comput. Soc. Conf. Comput. Vis. Pattern Recognit.* 2403–2412 (2018) doi:10.1109/CVPR.2018.00255.

6. He, K., Zhang, X., Ren, S. & Sun, J. Deep residual learning for image recognition. in *Proceedings of the IEEE Computer Society Conference on Computer Vision and Pattern Recognition* (2016). doi:10.1109/CVPR.2016.90.

7. Jocher, G. *et al.* ultralytics/yolov5: v2.0. *GitHub* (2020).

8. Russakovsky, O. *et al.* ImageNet Large Scale Visual Recognition Challenge. *Int. J. Comput. Vis.* **115**, 211–252 (2015).

9. Zhang, Y., Wang, C., Wang, X., Zeng, W. & Liu, W. FairMOT: On the Fairness of Detection and Re-identification in Multiple Object Tracking. *Int. J. Comput. Vis.* (2021) doi:10.1007/s11263-021-01513-4.

10. Ian Goodfellow, Yoshua Bengio, A. C. Deep Learning Book. *Deep Learn.* (2015) doi:10.1016/B978-0-12-391420-0.09987-X.

11. Shen, M. *et al.* A deep learning based automatic defect analysis framework for In-situ TEM ion irradiations. *Comput. Mater. Sci.* (2021) doi:10.1016/j.commatsci.2021.110560.

12. He, K., Zhang, X., Ren, S. & Sun, J. Delving deep into rectifiers: Surpassing human-level performance on imagenet classification. in *Proceedings of the IEEE International Conference on Computer Vision* (2015). doi:10.1109/ICCV.2015.123.

13. Glorot, X. & Bengio, Y. Understanding the difficulty of training deep feedforward neural networks. **9**, 249–256 (2010).

14. Doll, P., Girshick, R. & Noordhuis, P. Accurate, large minibatch SGD. *Arxiv* (2017).

15. Qiao, S., Wang, H., Liu, C., Shen, W. & Yuille, A. Weight standardization. *arXiv* (2019).

16. Smith, L. N. Cyclical learning rates for training neural networks. in *Proceedings - 2017 IEEE Winter Conference on Applications of Computer Vision, WACV 2017* (2017). doi:10.1109/WACV.2017.58.

17. NESTEROV, Y. A method for unconstrained convex minimization problem with the rate of convergence o(1/k^2). *Dokl. AN USSR* (1983).

18. Kingma, D. P. & Ba, J. L. Adam: A method for stochastic optimization. in *3rd International Conference on Learning Representations, ICLR 2015 - Conference Track Proceedings* (2015).

19. Duchi, J., Hazan, E. & Singer, Y. Adaptive subgradient methods for online learning and stochastic optimization. *J. Mach. Learn. Res.* (2011).

20. Johny, D. C. *et al.* ADADELTA: An Adaptive Learning Rate Method. *IEEE Access* (2018).

21. Hastie, T. T. The Elements of Statistical Learning Second Edition. *Math. Intell.* (2017).

22. Zheng, S., Song, Y., Leung, T. & Goodfellow, I. Improving the robustness of deep neural networks via stability training. *Proc. IEEE Comput. Soc. Conf. Comput. Vis. Pattern Recognit.* **2016**-**Decem**, 4480–4488 (2016).

23. Lin, T. Y. *et al.* Microsoft COCO: Common objects in context. in *Lecture Notes in Computer Science (including subseries Lecture Notes in Artificial Intelligence and Lecture Notes in Bioinformatics)* (2014). doi:10.1007/978-3-319-10602-1_48.

24. Paszke, A. *et al.* Automatic differentiation in PyTorch. in *Advances in Neural Information Processing Systems 32* (2019).

25. Everingham, M., Van Gool, L., Williams, C. K. I., Winn, J. & Zisserman, A. The pascal visual object classes (VOC) challenge. *Int. J. Comput. Vis.* **88**, 303–338 (2010).

26. Bernardin, K. & Stiefelhagen, R. Evaluating multiple object tracking performance: The CLEAR MOT metrics. *Eurasip J. Image Video Process.* (2008) doi:10.1155/2008/246309.

27. Ristani, E., Solera, F., Zou, R., Cucchiara, R. & Tomasi, C. Performance measures and a data set for multi-target, multi-camera tracking. in *Lecture Notes in Computer Science (including subseries Lecture Notes in Artificial Intelligence and Lecture Notes in Bioinformatics)* (2016). doi:10.1007/978-3-319-48881-3_2.

28. Karson, M. Handbook of Methods of Applied Statistics. Volume I: Techniques of Computation Descriptive Methods, and Statistical Inference. Volume II: Planning of Surveys and Experiments. I. M. Chakravarti, R. G. Laha, and J. Roy, New York, John Wiley; 1967, $9.00. *J. Am. Stat. Assoc.* **63**, 1047–1049 (1968).

29. Porter, F. C. Testing Consistency of Two Histograms. 1–35 (2008).

30. Sullivan, G. M. & Feinn, R. Using Effect Size—or Why the P Value Is Not Enough . *J. Grad. Med. Educ.* **4**, 279–282 (2012).

31. Cohen, J. *Statistical Power Analysis for the Behavioral Sciences*. (Taylor & Francis Group, 1988).
